# Metabolic Modeling and Bidirectional Culturing of Two Gut Microbes Reveal Cross-Feeding Interactions and Protective Effects on Intestinal Cells

**DOI:** 10.1128/msystems.00646-22

**Published:** 2022-08-25

**Authors:** Belén Hirmas, Naschla Gasaly, Guillermo Orellana, Marco Vega-Sagardía, Pedro Saa, Martín Gotteland, Daniel Garrido

**Affiliations:** a Department of Chemical and Bioprocess Engineering, School of Engineering, Pontificia Universidad Católica de Chile, Santiago, Chile; b Laboratory of Innate Immunity, Program of Immunology, Institute of Biomedical Sciences, Faculty of Medicine, Universidad de Chile, Santiago, Chile; c Department of Nutrition, Faculty of Medicine, University of Chile, Santiago, Chile; d Institute for Mathematical and Computational Engineering, Pontificia Universidad Católica de Chile, Santiago, Chile; University of California, San Francisco

**Keywords:** cross-feeding, genome-scale metabolic models, butyrate, prebiotics

## Abstract

The gut microbiota is constituted by thousands of microbial interactions, some of which correspond to the exchange of metabolic by-products or cross-feeding. Inulin and xylan are two major dietary polysaccharides that are fermented by members of the human gut microbiota, resulting in different metabolic profiles. Here, we integrated community modeling and bidirectional culturing assays to study the metabolic interactions between two gut microbes, *Phocaeicola dorei* and Lachnoclostridium symbiosum, growing in inulin or xylan, and how they provide a protective effect in cultured cells. *P. dorei* (previously belonging to the *Bacteroides* genus) was able to consume inulin and xylan, while *L. symposium* only used certain inulin fractions to produce butyrate as a major end product. Constrained-based flux simulations of refined genome-scale metabolic models of both microbes predicted high lactate and succinate cross-feeding fluxes between *P. dorei* and *L. symbiosum* when growing in each fiber. Bidirectional culture assays in both substrates revealed that *L. symbiosum* growth increased in the presence of *P. dorei*. Carbohydrate consumption analyses showed a faster carbohydrate consumption in cocultures compared to monocultures. Lactate and succinate concentrations in bidirectional cocultures were lower than in monocultures, pointing to cross-feeding as initially suggested by the model. Butyrate concentrations were similar across all conditions. Finally, supernatants from both bacteria cultured in xylan in bioreactors significantly reduced tumor necrosis factor-α-induced inflammation in HT-29 cells and exerted a protective effect against the TcdB toxin in Caco-2 epithelial cells. Surprisingly, this effect was not observed in inulin cocultures. Overall, these results highlight the predictive value of metabolic models integrated with microbial culture assays for probing microbial interactions in the gut microbiota. They also provide an example of how metabolic exchange could lead to potential beneficial effects in the host.

**IMPORTANCE** Microbial interactions represent the inner connections in the gut microbiome. By integrating mathematical modeling tools and microbial bidirectional culturing, we determined how two gut commensals engage in the exchange of cross-feeding metabolites, lactate and succinate, for increased growth in two fibers. These interactions underpinned butyrate production in cocultures, resulting in a significant reduction in cellular inflammation and protection against microbial toxins when applied to cellular models.

## INTRODUCTION

The gut microbiota is represented by a complex network of microorganisms occupying different niches in the human gut (1, 2). Their abundances change dynamically according to age and perturbations such as antibiotic administration (3–5). The composition and activity of the gut microbiota are influenced by the diet, especially by molecules that escape digestion in the small intestine, rendering them available for fermentation in the large intestine (6).

Soluble dietary fibers are fermented by the gut microbiota and include structurally diverse polysaccharides found in plant cell walls. Inulin is a class of fructan, i.e., a polymer of fructose linked by β-2,1 bonds with a degree of polymerization of 10 to 30 (7). Inulin is a well-reported prebiotic, promoting the growth of *Bifidobacterium* species in *in vivo* studies (8). However, other species from the *Bacteroides* and *Lachnospiraceae* groups can also ferment inulin (9, 10). Additionally, inulin shapes the gut microbiota decreasing the number of undesirable microorganisms such as Bilophila wadsworthia (8).

Other fermentable fibers include pectin, resistant starch, and xylan (11). Xylan is the second most abundant component in plant cell walls, especially in grains and seeds (12). It is composed of linear chains of xylose from 10 to 100 monomers, with side chains consisting of glucuronic acid, acetyl groups, and arabinose (12). The mechanisms for xylan degradation and fermentation have been described in certain *Bacteroides* species such as *B. ovatus* (13). Both inulin and xylan are fermented by complex networks of microorganisms (14), where some species act as degraders accessing the complex linkages in these molecules. This process releases intermediate degradation products such as smaller carbohydrate chains, short-chain fatty acids (SCFAs) such as acetate, propionate, and butyrate, and other organic acids such as lactate and succinate (15–17). These SCFAs are produced in a 3:1:1 ratio, reaching fecal concentrations of 70 to 140 mM (18). Butyrate is the primary energy source for the colonocytes (19). In addition, SCFAs have been increasingly involved in physiological host responses (16, 18). Indeed, butyrate is a critical epigenetic regulator inhibiting histone deacetylases in colonocytes, suppressing proinflammatory pathways (20).

Gut microorganisms have evolved sophisticated mechanisms for interacting with each other, for example, by exploitative competition (21) or the metabolic exchange of by-products (cross-feeding) (22). Metabolic cross-feeding is a common interaction in the gut microbiota (23). Polysaccharide remnants released by certain *Bacteroides* or *Bifidobacterium* species can be used by other species (24, 25). Other exchanged molecules are fermentation by-products such as acetate, lactate, and succinate (26–28). Butyrate-producing bacteria such as *Roseburia* sp., Faecalibacterium prausnitzii, and Eubacterium rectale preferentially use these organic acids as a carbon source for butyrate production (29, 30). Anaerostipes caccae releases 5-fold more butyrate from lactate than glucose (31). Among four main metabolic pathways leading to butyrate production in the gut microbiota, the 4-aminobutyrate pathway in certain clostridia has been less studied (32).

Cross-feeding interactions are critical to understand and eventually predict the impact of dietary fibers on the gut microbiota. The availability of genome and metagenome data as well as increasing biochemical information for microorganisms has enabled the construction of Genome-Scale Metabolic Models (GSMMs) (33, 34). These model structures can be employed to explore the potential of cellular metabolism. Particularly in the case of microbial communities, they can readily suggest metabolic interactions between members of the microbiome (35, 36). Metabolic modeling and literature reconstructions predict a dense cross-feeding network between dietary substrates and, most notably, exploitative competition and metabolic cross-feeding as the two most common interactions in the gut microbiota (37–39). Constructing accurate GSMMs for poorly studied gut microbes remains a challenge as there are several knowledge gaps in their genetic makeup, pathways, and overall metabolic capabilities (40, 41). However, comprehensive metabolic network reconstruction resources such as AGORA and BIGG, accompanied by constraint-based modeling methods and algorithms, have proved effective for suggesting and understanding complex microbial interactions in the gut microbiota (36, 42, 43). Among the most useful algorithms, SteadyCom (44) stands out as a scalable linear optimization method for predicting microbial composition assuming a fast and fixed community growth, which has been theoretically proven to hold true under balanced growth (45). In addition, this computation predicts the required metabolic exchanges to sustain such growth, thereby revealing likely cross-feeding interactions under growth optimality. Importantly, this framework can be readily combined with other constraint-based methods such as Flux Variability Analysis (FVA) (46) and even Monte Carlo flux sampling (47), for the assessment of the metabolic flexibility under a defined community state.

Previous studies have shown that two gut commensals, *Phocaeicola dorei* (previously assigned to the *Bacteroides* genus) and Lachnoclostridium symbiosum, are key species in butyrate production within a 15-species synthetic consortium (48), showing an anti-inflammatory effect in human intestinal epithelial cells when supplemented with the culture supernatant (49). Yet, the cross-feeding mechanisms underpinning microbial growth and butyrate production remain poorly understood, and metabolic modeling could provide significant insights into microbial interactions leading to butyrate production. Here, we used a combination of metabolic modeling and bidirectional cross-feeding assays to unravel the metabolic interactions supporting their growth on different dietary fibers (inulin and xylan) in cocultures. The protective effect of their metabolic production profile in coculture was again demonstrated by the anti-inflammatory effect exerted on intestinal epithelial cells.

## RESULTS

**FIG 1:**
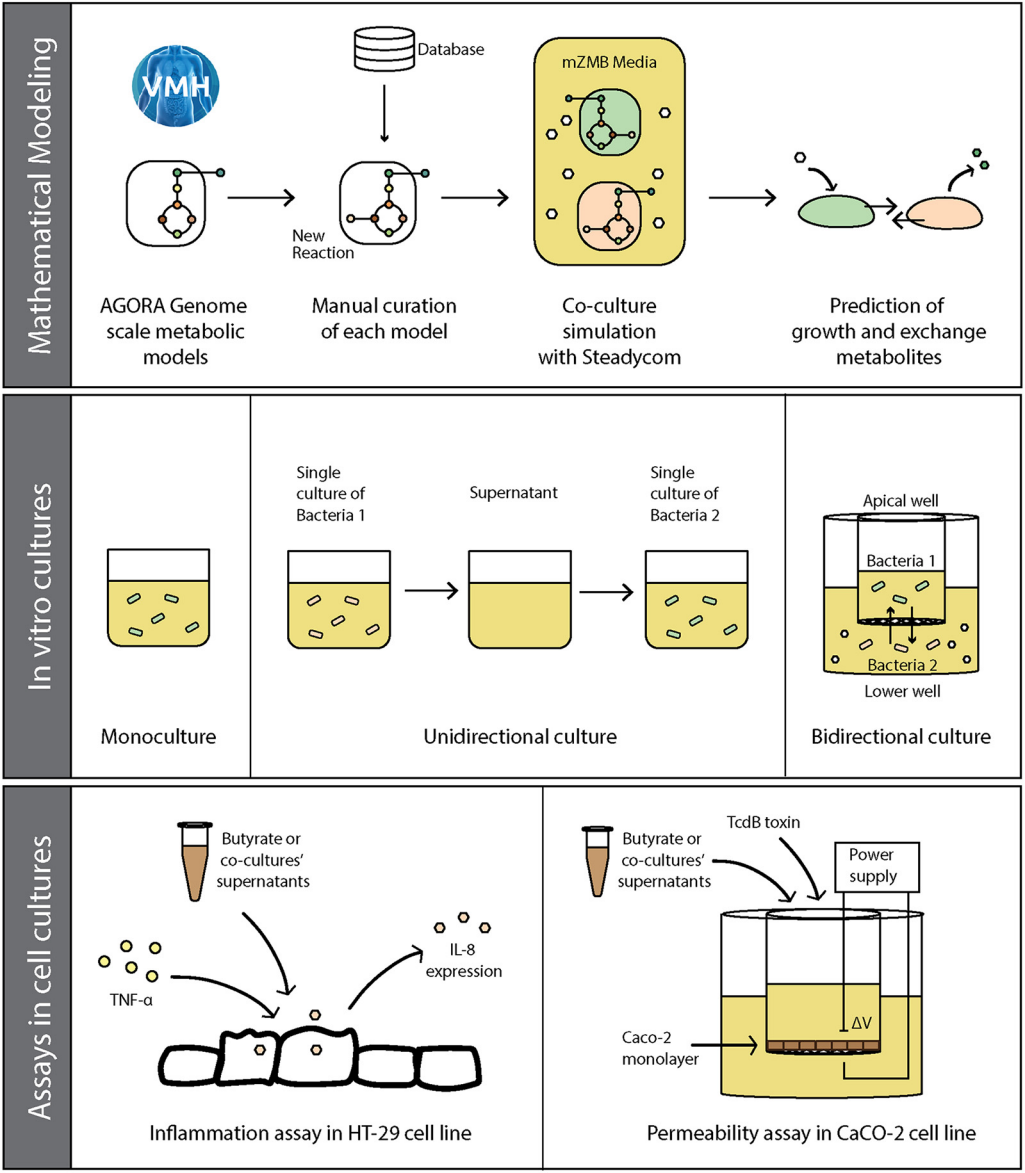
General integrated workflow for identifying cross-feeding interactions. Initial GSMMs for *P. dorei* and *L. symbiosum* were obtained from the AGORA database and manually curated with literature information, especially for inulin and xylan utilization reactions. Their metabolic interactions on these substrates were simulated using SteadyCom and Monte Carlo random sampling. Both bacteria were cultured in bidirectional and monodirectional assays and screened for substrate consumption, SCFA production, and changes in gene expression. Finally, the effect of their supernatants was evaluated in cell cultures in markers of inflammation and cell permeability.

A summary of the approaches followed in this study is depicted in Fig. 1. First, metabolic reconstructions were employed to simulate *in silico* the combined growth and metabolite exchange by *P. dorei* and *L. symbiosum* during inulin and xylan utilization. Then, *in vitro* cocultures were performed to determine the potential for simultaneous bidirectional exchange of metabolites. Finally, both microorganisms were cultured in bioreactors and their spent supernatant was applied to intestinal cells to evaluate their protective effect. Our main results are next presented following the aforementioned workflow.

### Adaptation of genome-scale metabolic network reconstructions of *P. dorei* and *L. symbiosum* for flux simulations under inulin and xylan utilization.

To probe possible mechanisms of metabolic cross-feeding between the studied microorganisms, genome-scale network reconstructions (GENREs) of *P. dorei* 5_1_36 and *L. symbiosum* WAL16173 were obtained from the AGORA v3.0 database and adapted before their use. Reconstructions were manually refined to include known information about their capabilities for degrading inulin and xylan. For instance, inulin and xylan have different chain lengths, and the average carbon amounts were normalized to allow proper comparison (Table S1 in the supplemental material). The capabilities of the curated model reconstructions were assessed by comparing the predicted and experimental substrate utilization and metabolic production of various compounds reported in the literature (Tables S2 to S3 and S5 to S6). Overall, the models displayed high accuracy in these tests with *F*-score values (max. 1.0) of 0.70 for *L. symbiosum* and 0.83 for *P. dorei* (Table S4, respectively). As a final measure of quality, both models were evaluated with the MEMOTE tool (50) (refer to Materials and Methods) displaying a score above 92% in the consistency test (Table S7), pointing to a reasonably high model quality in both cases. These models were subsequently employed to simulate the metabolic behavior of the studied consortium.

10.1128/msystems.00646-22.4 TABLE S1 Modifications introduced to the AGORA reconstructions of *P. dorei* and *L. symbiosum*. Download Table S1, PDF file, 0.7 MB. Copyright © 2022 Hirmas et al. 2022 Hirmas et al. https://creativecommons.org/licenses/by/4.0/ This content is distributed under the terms of the Creative Commons Attribution 4.0 International license.

10.1128/msystems.00646-22.5 TABLE S2 Experimental evidence and model prediction of substrate consumption for *L. symbiosum*. Download Table S2, PDF file, 0.3 MB. Copyright © 2022 Hirmas et al. 2022 Hirmas et al. https://creativecommons.org/licenses/by/4.0/ This content is distributed under the terms of the Creative Commons Attribution 4.0 International license.

10.1128/msystems.00646-22.6 TABLE S3 Experimental evidence and model prediction of fermentation products in *L. symbiosum*. Download Table S3, PDF file, 0.3 MB. Copyright © 2022 Hirmas et al. 2022 Hirmas et al. https://creativecommons.org/licenses/by/4.0/ This content is distributed under the terms of the Creative Commons Attribution 4.0 International license.

10.1128/msystems.00646-22.7 TABLE S4 *L. symbiosum* and *P. dorei* model evaluation results. Download Table S4, PDF file, 0.3 MB. Copyright © 2022 Hirmas et al. 2022 Hirmas et al. https://creativecommons.org/licenses/by/4.0/ This content is distributed under the terms of the Creative Commons Attribution 4.0 International license.

10.1128/msystems.00646-22.8 TABLE S5 Experimental evidence and model prediction of substrate consumption of *P. dorei*. Download Table S5, PDF file, 0.3 MB. Copyright © 2022 Hirmas et al. 2022 Hirmas et al. https://creativecommons.org/licenses/by/4.0/ This content is distributed under the terms of the Creative Commons Attribution 4.0 International license.

10.1128/msystems.00646-22.9 TABLE S6 Experimental evidence and model prediction of fermentation products in *P. dorei*. Download Table S6, PDF file, 0.3 MB. Copyright © 2022 Hirmas et al. 2022 Hirmas et al. https://creativecommons.org/licenses/by/4.0/ This content is distributed under the terms of the Creative Commons Attribution 4.0 International license.

10.1128/msystems.00646-22.10 TABLE S7 MEMOTE model evaluation results. Download Table S7, PDF file, 0.3 MB. Copyright © 2022 Hirmas et al. 2022 Hirmas et al. https://creativecommons.org/licenses/by/4.0/ This content is distributed under the terms of the Creative Commons Attribution 4.0 International license.

### Flux simulations of *L. symbiosum* and *P. dorei* cocultures revealed tight lactate and succinate cross-feeding under inulin and xylan utilization.

We first aimed to identify which metabolites are predicted to be exchanged within the community under the assumption of optimal (maximum) balanced growth on the two fibers. For this task, the experimentally observed community growth rates on inulin (0.35 h^−1^) and xylan (0.09 h^−1^) were constrained in the models (Fig. S1 in the supplemental material). The minimal fiber consumption rates of inulin and xylan were computed using the SteadyCom formulation. The last calculation is equivalent to determining the maximum community growth under a fixed inulin or xylan consumption acting as main carbon sources. We note that the experimentally determined community growth rates are consistent with the balanced growth assumption of the SteadyCom formulation, as both the community growth rate and microbial abundances remained constant over time (midexponential growth phase) for this calculation.

10.1128/msystems.00646-22.1 FIG S1 Cocultures of *P. dorei* and *L. symbiosum* in bioreactors. (Top) Growth curves (OD_600_) for inulin and xylan. Experimentally observed community growth rates at midexponential phase growing on inulin and xylan were 0.35 h^−1^ and 0.09 h^−1^, respectively (12 h ≤ t ≤ 24 h). (Middle) Relative abundance for *L. symbiosum* and *P. dorei* in inulin (left) and xylan (right). In the same period for the community growth calculation, the microbial abundances were stable and were 0.159 on inulin and 0.091 on xylan for *P. dorei*, and 0.841 on inulin and 0.909 on xylan for *L. symbiosum*. (Bottom) Concentrations of SCFAs and lactate in bioreactors at the end of each run in inulin (left) or xylan (right). Pd, *P. dorei*; Ls, *L. symbiosum*. Download FIG S1, TIF file, 0.1 MB. Copyright © 2022 Hirmas et al. 2022 Hirmas et al. https://creativecommons.org/licenses/by/4.0/ This content is distributed under the terms of the Creative Commons Attribution 4.0 International license.

**FIG 2:**
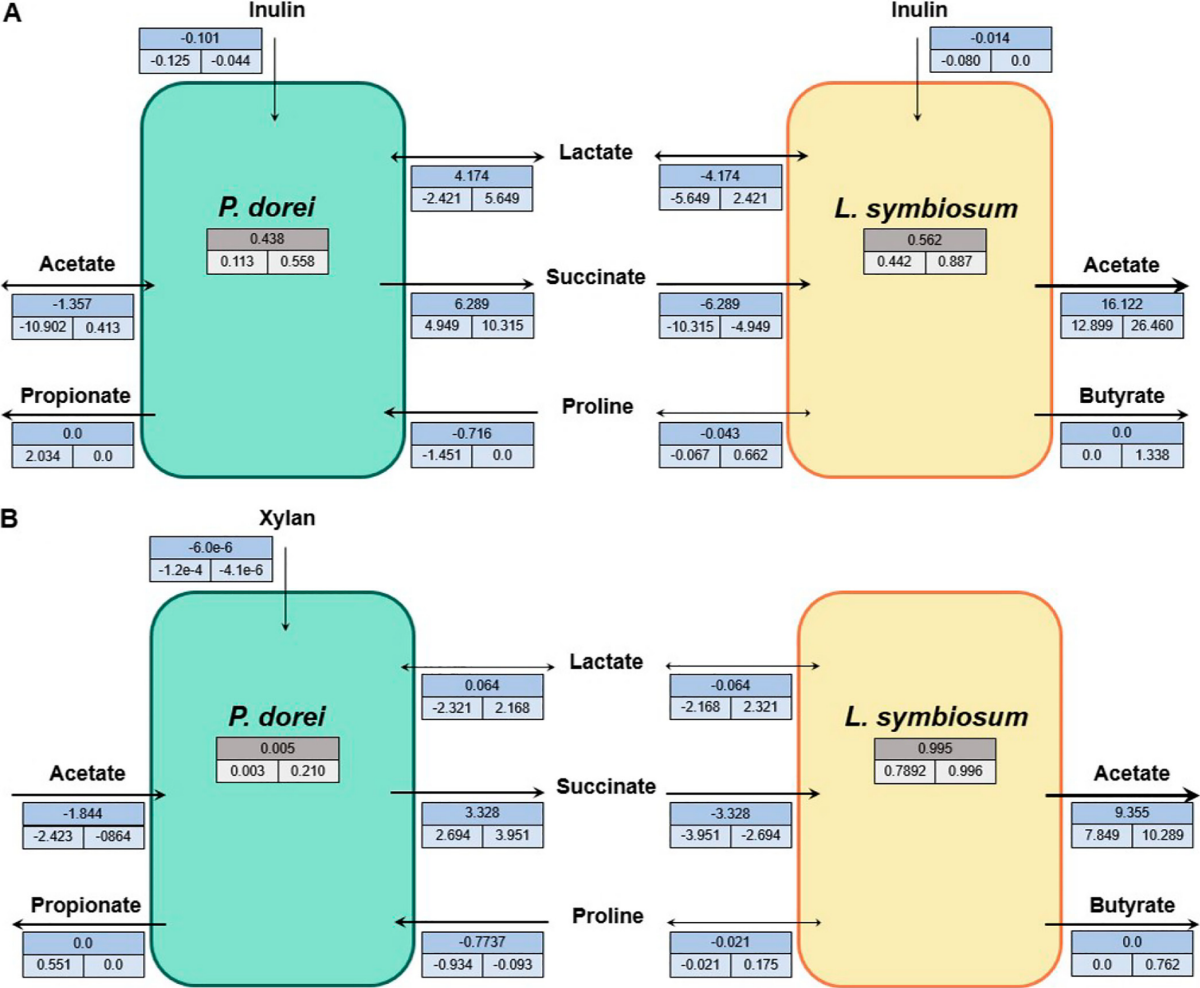
Flux exchanges of major metabolites between *P. dorei* and *L. symbiosum* predicted by SteadyCom under (sub)optimal growth on inulin and xylan. Experimental community growth rates observed on inulin (A) and xylan (B) were constrained in the models (Fig. S1 in the supplemental material), and the minimal fiber consumption rates of inulin and xylan were computed using SteadyCom. The resulting flux distribution represents the optimal flux distribution growing on each fiber, and it is shown in the upper value of each box. Suboptimal growth under each condition was also simulated by constraining the previous community growth rates allowing for a higher fiber consumption. Minimum and maximum flux values for each variable are shown in the lower values of each box. Finally, blue boxes represent absolute fluxes (in mmol/h), whereas as gray boxes represent microbial abundances. Arrow heads represent the direction of flux exchanges. Arrow thickness is proportional to the absolute flux value.

Under optimal community growth on each fiber, *L. symbiosum* was predicted to reach a greater abundance than *P. dorei*, which was substantially higher when growing on xylan (Fig. 2). Under inulin growth, *P. dorei* showed an approximately 7.2-fold higher inulin consumption than *L. symbiosum* (Fig. 2A), whereas only *P. dorei* consumed xylan in the other scenario (Fig. 2B). This could be explained by *L. symbiosum* targeting small fractions or showing a lower efficiency toward inulin utilization. Notably, flux simulations under optimal fiber growth showed no butyrate nor propionate production, albeit there was a relatively high exchange of succinate, lactate, and acetate between *P. dorei* and *L. symbiosum* (Fig. 2). This exchange supported a faster growth of *L. symbiosum* in the presence of *P. dorei*, which was later confirmed experimentally (see next section).

To explore the metabolic flexibility in the community, we simulated suboptimal utilization of both carbon sources by allowing a higher inulin or xylan consumption enabling a 5% higher community growth rate under each condition and then again fixing the latter to the experimental values. Application of FVA in these scenarios revealed that the abundances of *P. dorei* and *L. symbiosum* ranged, respectively, from 0.113 to 0.558 and 0.442 to 0.887 under inulin growth and from 0.003 to 0.210 and 0.789 to 0.996 under xylan utilization (Fig. 2). Notably, the predicted abundance ranges contained the observed values for *P. dorei* (0.159 on inulin and 0.091 on xylan, Fig. S1) and *L. symbiosum* (0.841 on inulin and 0.909 on xylan, Fig. S1), allowing the production of propionate and butyrate. More importantly, analysis of the flux variabilities for metabolic exchanges pointed to a unidirectional cross-feeding of succinate from *P. dorei* to *L. symbiosum* under both conditions (as indicated by the negative flux values in *P. dorei* and positive values in *L. symbiosum*) and a bidirectional cross-feeding of acetate and lactate (Fig. 2).

**FIG 3:**
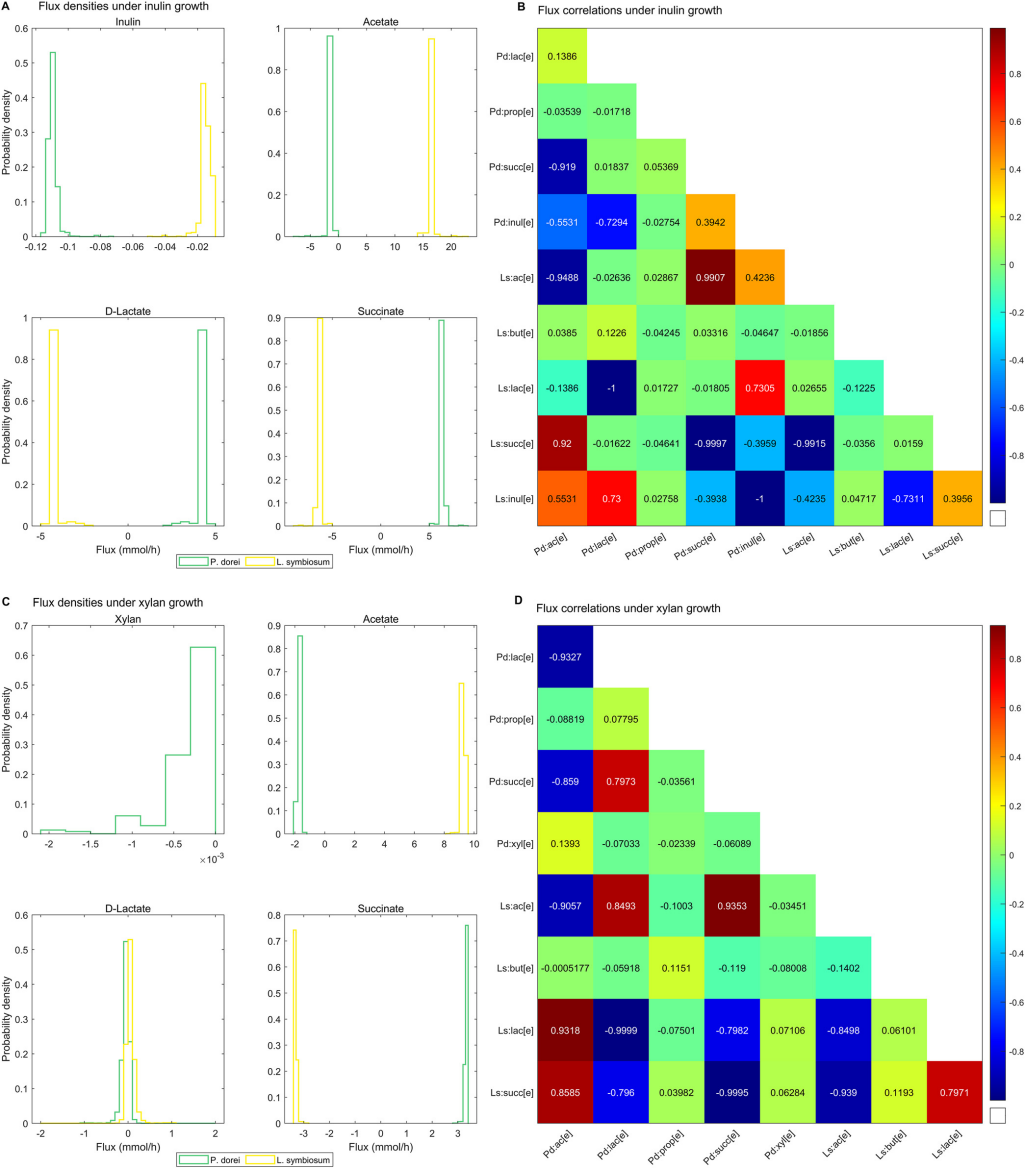
Metabolic coupling of exchanged metabolites in cocultures of *P. dorei* and *L. symbiosum* predicted by random sampling of the flux solution space. (A and C) Density plots for exchanged metabolites during simulations of inulin (A) or xylan (C) consumption by *P. dorei* and *L. symbiosum*. Plots represent 200,000 flux solutions that match the experimentally observed community growth rate in the suboptimal fiber consumption scenario. (B and D) Pairwise correlation between metabolite exchanges under inulin (B) and xylan (D) growth.

To further investigate these interactions, Monte Carlo sampling was employed to explore the distribution and coupling between exchanged metabolites under suboptimal conditions (Fig. 3). Under inulin growth, the *P. dorei* lactate flux exchange was significantly greater than zero, i.e., it was produced (*P* < 0.01, Wilcoxon rank sum test), while it was significantly lower than zero for *L. symbiosum*, i.e., it was consumed (*P* < 0.01, Wilcoxon rank sum test) (Fig. 3A). This was also supported by the perfect anticorrelation (−1.0) between the exchanges of both microbes (Fig. 3B). The case of acetate was the opposite; *P. dorei* consumed the acetate produced by *L. symbiosum*, albeit with a slightly lower absolute (negative) correlation (−0.95, Fig. 3B). On the other hand, under xylan growth, only acetate followed the previous trend (Fig. 3C). Lactate could be either produced or consumed by *L. symbiosum* or *P. dorei*, although it required full coupling (anticorrelation of −1.0) between the exchanges of both microbes (Fig. 3D). Finally, while butyrate production by *L. symbiosum* showed the highest absolute cross-feeding correlation with lactate produced by *P. dorei* under inulin utilization (0.12, Fig. 3B), succinate showed the highest absolute cross-feeding correlation (0.12, Fig. 3D) under xylan utilization. Overall, these results suggest that both lactate and succinate are actively exchanged and play important roles in community growth and butyrate production under the studied conditions.

### Bidirectional and unidirectional culture assays suggest synergistic effects causing increased growth in cocultures.

The metabolic exchange between *P. dorei* and *L. symbiosum* was experimentally assessed in bidirectional assays. In this setup, microorganisms are cultured either in the bottom well or top insert of a Transwell plate, separated by a 0.1-μm membrane filter. The filter allows the exchange of small and medium-sized metabolites that reach chemical equilibrium.

**FIG 4:**
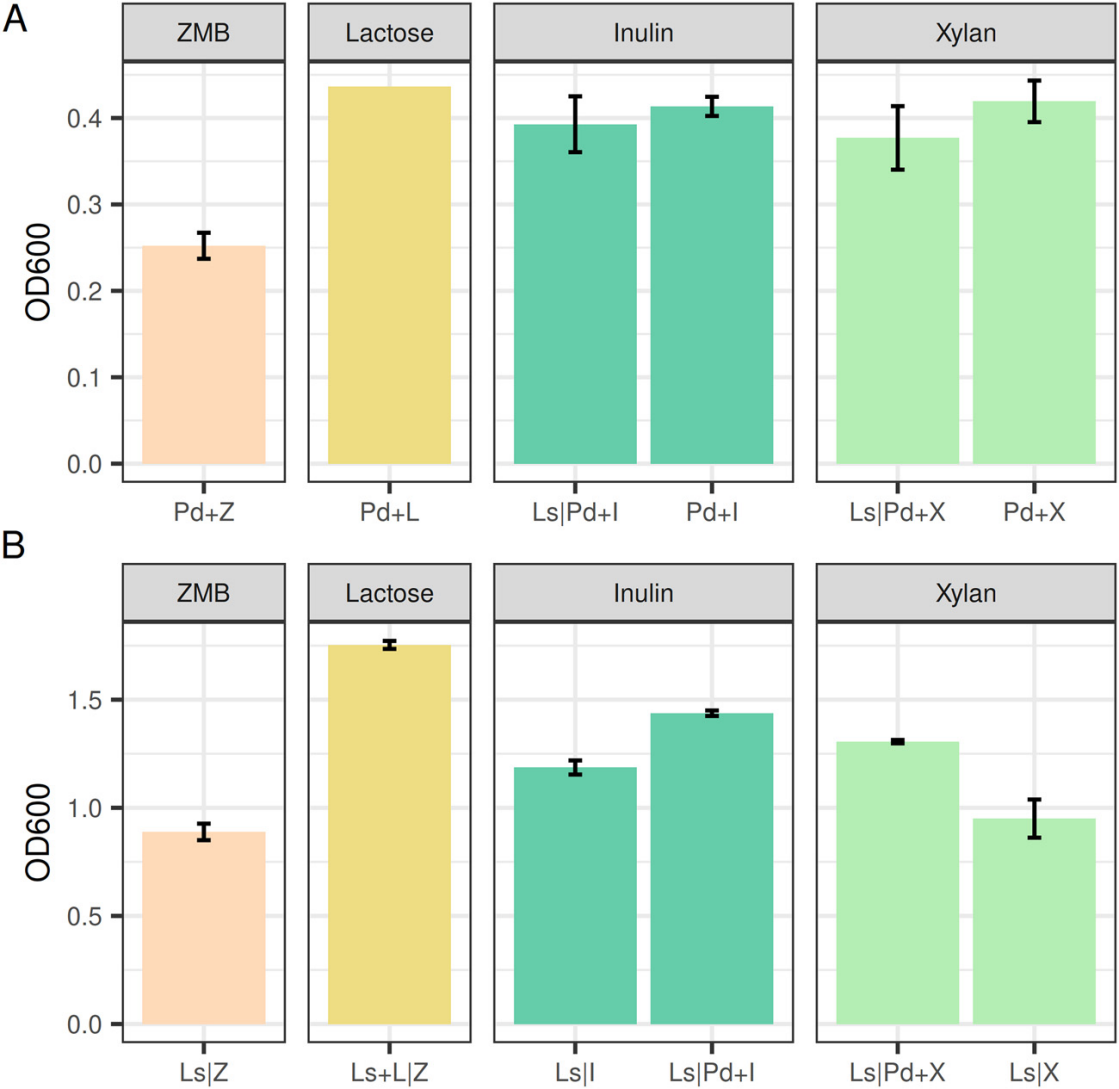
Maximum growth value (OD_600_) reached by *P. dorei* (A) and *L. symbiosum* (B) in bidirectional assays. Bacteria were cultured in inulin (I) or xylan (X). Lactose (L) was used as positive control, and Z represents medium with no carbon source. Vertical line in the *x* axis (|) represents the separation by the membrane.

*P. dorei* reached moderate biomass concentration values in inulin and xylan, similar to lactose (Fig. 4A). The presence of *L. symbiosum* did not significantly alter the growth of *P. dorei* in this assay (*P* > 0.05 by *t* test). On the other hand, *L. symbiosum* presented a moderate basal growth, which correlates with the ability of clostridia to ferment amino acids (51). However, as opposed to *P. dorei*, *L. symbiosum* reached higher growth in the presence of *P. dorei* in both xylan and inulin (Fig. 4B; *P* < 0.05 by *t* test). Particularly, *L. symbiosum* growth using inulin was higher than the basal, suggesting partial fructans utilization (Fig. 4 and Fig. S2; *P* < 0.05 by *t* test).

10.1128/msystems.00646-22.2 FIG S2 Cellular growth of *P. dorei* and *L. symbiosum* in bidirectional assays and unidirectional setup under different carbon sources. Cellular growth of *P. dorei* and *L. symbiosum* in bidirectional assays (*P. dorei* [A] and *L. symbiosum* [C]) and in unidirectional or monocultures (*P. dorei* [B] and *L. symbiosum* [D]). In each case, biomass measurements (OD_600_) were collected at 0, 24, and 48 h. Each condition represents a biological triplicate, and error bars denote one standard deviation. Pd, *P. dorei*; Ls, *L. symbiosum*; L, lactose; I, inulin; X, xylan; Z, mZMB culture medium. Download FIG S2, TIF file, 0.6 MB. Copyright © 2022 Hirmas et al. 2022 Hirmas et al. https://creativecommons.org/licenses/by/4.0/ This content is distributed under the terms of the Creative Commons Attribution 4.0 International license.

In unidirectional experiments, similar growth values were observed for *P. dorei* in inulin alone or using the spent supernatant from *L. symbiosum* in inulin (Fig. S2). Further, unidirectional *P. dorei* growth was comparable to its monoculture and when it used the supernatant of *L. symbiosum* cultured in xylan (Fig. S2; *P* > 0.05 by *t* test). This suggests that *P. dorei* does not see a major impact on its growth in the presence of *L. symbiosum*, probably consuming the spent substrates not used by *L. symbiosum*. Supernatants after 48 h of growth of *P. dorei* inoculated with inulin or xylan did not stimulate the growth of *L. symbiosum* in the unidirectional assay (Fig. S2; *P* > 0.05 by *t* test). This suggests that the growth increase of *L. symbiosum* in the presence of *P. dorei* depends on the synergistic growth of both microorganisms.

### Increased growth in cocultures is supported by faster fiber consumption.

**FIG 5:**
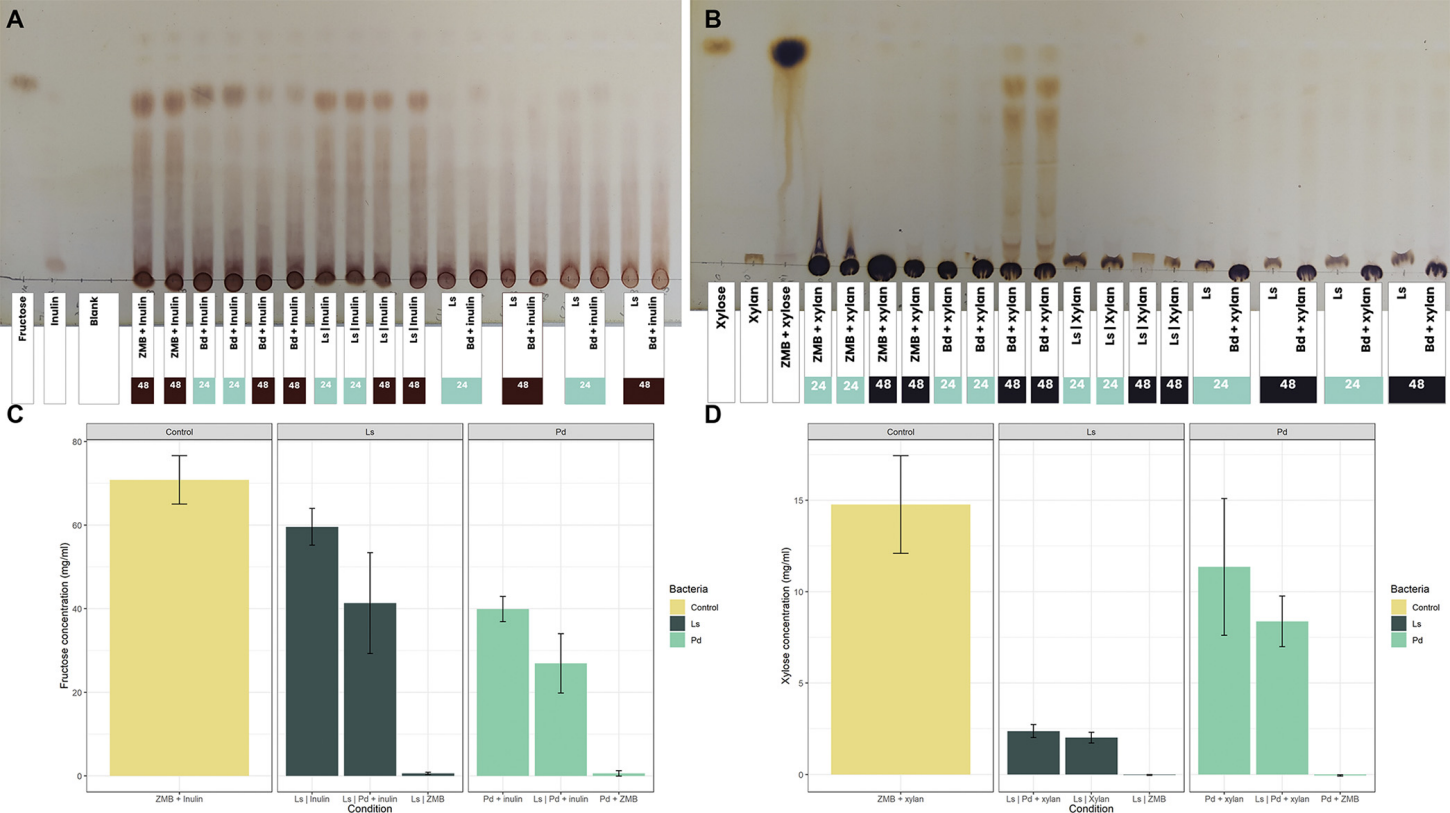
Carbohydrate analyses of coculture supernatants. Thin layer chromatography of all the conditions in mZMB with inulin (A) or xylan (B) in the bidirectional assays. Each rectangle represents one condition in the well of the bacteria (e.g., “Ls | Z” indicates the insert with *L. symbiosum*). Wider rectangles indicate first the insert and then the lower well. Samples of 24 h and 48 h were marked in different colors, while standards are colorless. (C) Fructose quantification in inulin supernatants using the phenol-sulfuric acid method after 48 h. (D) Xylose quantification of xylan supernatants after 48 h.

To better understand the mechanisms underpinning the higher growth in cocultures on inulin and xylan, we screened the supernatants from bidirectional assays for carbohydrate consumption (Fig. 5). For *P. dorei*, thin-layer chromatography (TLC) plates and carbohydrate quantification showed a reduction in total inulin concentration after 48 h (*P* < 0.05 by *t* test), especially the intermediate and smaller fractions. *L. symbiosum* also reduced the concentration of inulin in the medium, albeit to a lesser extent (Fig. 5A and C). The *P. dorei*-*L. symbiosum* coculture showed an important reduction in inulin concentration, larger than each individual consumption (Fig. 5C; *P* < 0.05 by *t* test). TLC showed that this pair consumed all inulin fractions after 48 h. These results indicate an accelerated consumption of inulin in coculture, reaching almost total consumption at 24 h.

*P. dorei* monoculture in xylan resulted only in partial substrate consumption after 48 h, leaving degradation products as shown by TLC (Fig. 5B and D). On the other hand, *L. symbiosum* was unable to degrade xylan. The coculture of both microorganisms showed a reduction in total carbohydrates in the medium (Fig. 5D; *P* < 0.05 by *t* test) but no visible degradation products (Fig. 5B). Xylan-derived glycans generated in monoculture might be consumed faster in coculture, as suggested by the reduction of carbohydrates (Fig. 5D).

### Metabolite exchanges in bidirectional culture assays are consistent with modeling predictions.

**FIG 6:**
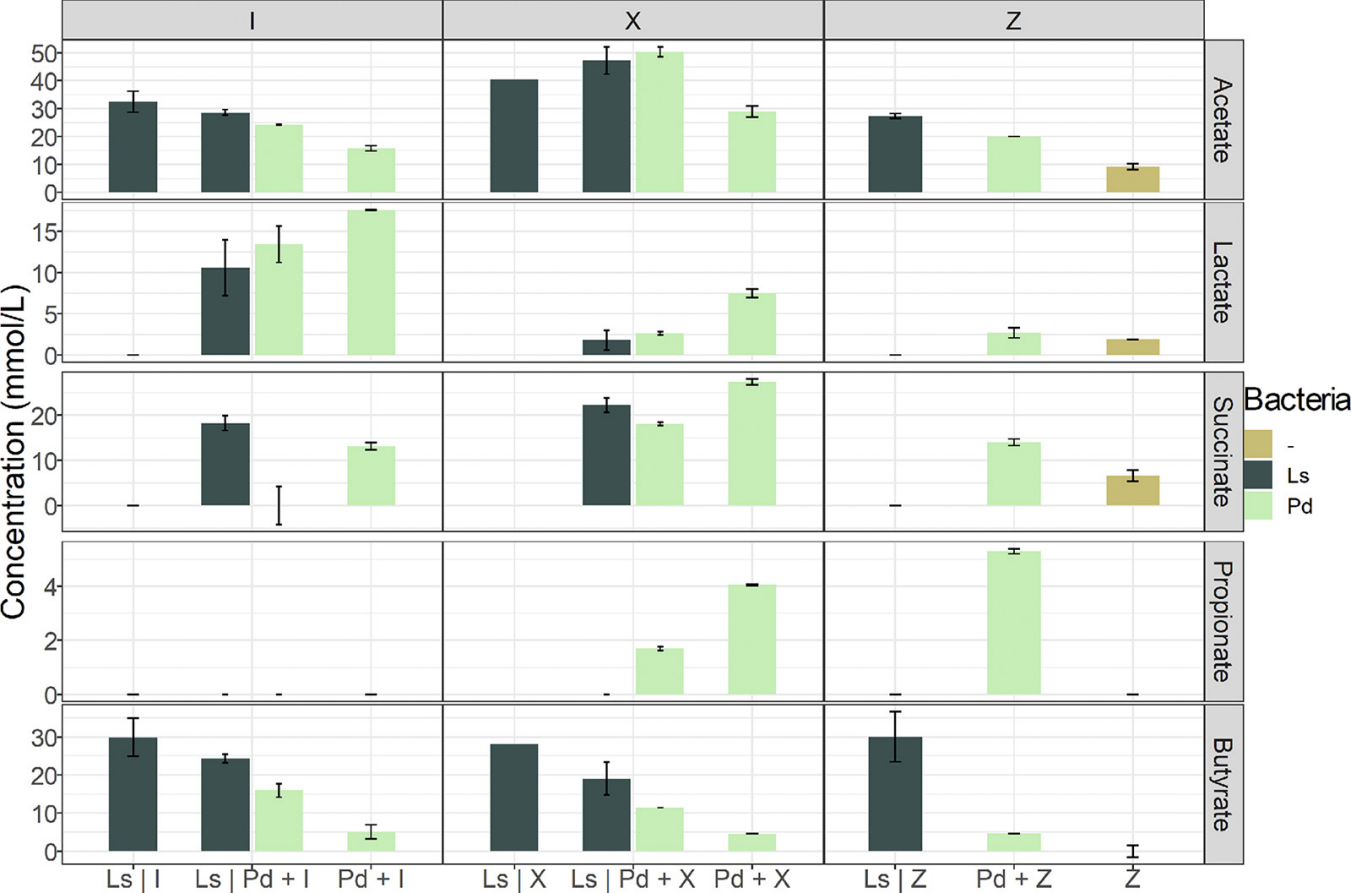
Concentrations of SCFAs and organic acids in bidirectional assays measured after 48 h. Acids are indicated in the right, and concentrations (mmol/liter) are in the left axis. Inulin (I) experiments include Ls as monoculture, Ls | Pd + I as the coculture, and Pd + I as *P. dorei* monoculture. Xylan experiments (X) follow the same terminology. Z bars indicate the controls with no culture medium.

SCFAs, lactate, and succinate concentrations were quantified in bidirectional assays and compared to the amounts observed in monocultures (Fig. 6). *L. symbiosum* monoculture supernatants did not contain lactate in any sample. Single cultures of *P. dorei* produced 17 mM lactate in inulin and 7 mM lactate in xylan after 48 h. The presence of *L. symbiosum* reduced lactate amounts in both compartments (*P* < 0.05 by *t* test). This corresponds to an approximate reduction of lactate of 15% (in inulin) and 80% (in xylan) with respect to total lactate produced by *P. dorei* alone. Similarly, only *P. dorei* produced succinate. Its concentration was higher after growing in xylan than inulin after 48 h (Fig. 6; *P* > 0.05 by *t* test). A reduction in succinate was also observed in xylan cocultures (*P* > 0.05 by *t* test), pointing to the presence of *L. symbiosum* as possibly being responsible (Fig. 6). These results are overall consistent with the previous modeling predictions.

Higher acetate production was observed in cocultures than in monocultures, both in inulin and xylan (Fig. 6; *P* < 0.05 by *t* test). Xylan utilization resulted in higher acetate concentrations, confirming previous modeling results. Propionate was produced by *P. dorei* at much lower concentrations in media with no carbon source or xylan, but not inulin (Fig. 6). Propionate produced by *P. dorei* was reduced by about half in the presence of *L. symbiosum*.

Finally, concentrations between 20 and 30 mM butyrate were detected in *L. symbiosum* monocultures in the tested carbon sources and control medium (Fig. 6). Butyrate concentrations in the *L. symbiosum* compartment were not modified by the presence of *P. dorei* in the basal compartment (*P* > 0.05). These results indicate that butyrate production is independent of the substrate under these conditions.

### Gene expression changes explain differential fiber utilization of *P. dorei* and *L. symbiosum* in cocultures.

**FIG 7:**
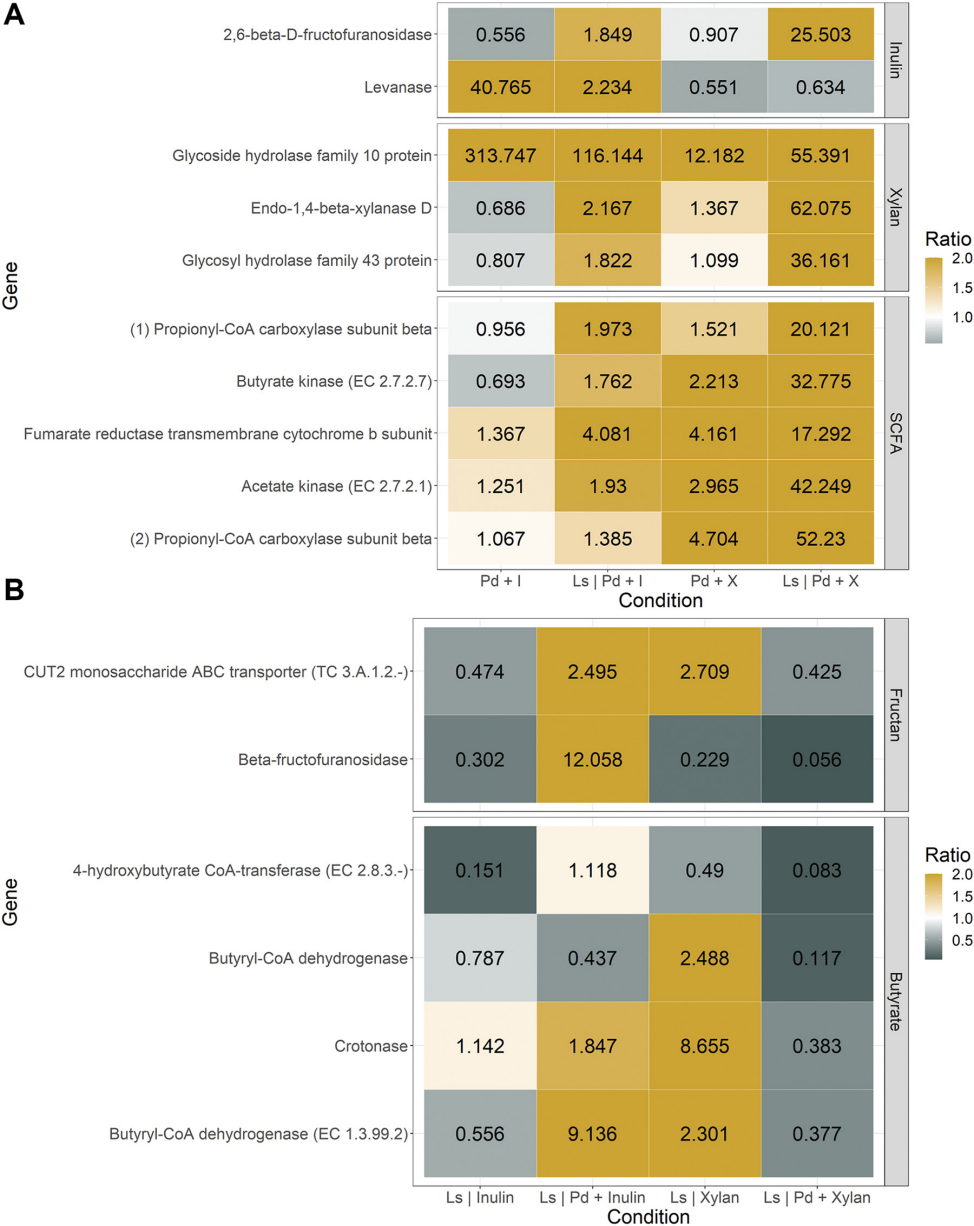
Relative expression of genes of interest of *P. dorei* and *L. symbiosum* growing on inulin and xylan. Each column represents the culture conditions, whereas the rows represent the corresponding gene expression fold change. Right labels indicate the pathways to which each gene belongs, while the number and color represent the expression ratio. Ratios over 1.5 and under 0.5 are colored as the limits of the ratio’s legend, indicating overexpression or repression.

Next, relative expression changes of relevant genes or pathways were analyzed in order to contrast the observed fiber utilization patterns. *P. dorei* monoculture in inulin showed the induction of a levanase (Fig. 7A). However, its expression was reduced in coculture, leading to an increase in the relative expression of a fructofuranosidase (Fig. 7A). Similar gene functions in *L. symbiosum* were induced in the presence of *P. dorei* in inulin (Fig. 7B). These results help to explain the higher inulin degradation observed in coculture compared to monocultures. In the presence of *L. symbiosum*, *P. dorei* showed an increase in the expression of three putative xylan-utilization genes, especially an endo-1-4-beta-xylanase (Fig. 7A). These observations support increased consumption of xylan in coculture.

Putative genes participating in SCFA production by *P. dorei* (such as acetate kinase) were more active in coculture in xylan. This correlates with the higher acetate production reported above. A reduction in butyrate-producing enzymes in *L. symbiosum* in the xylan coculture was found when comparing the growth in basal medium or *L. symbiosum* alone. Finally, genes potentially contributing to succinate and propionate production in the *P. dorei* genome were induced in xylan coculture correlating with concentration data (Fig. 7A).

### Supernatants of *P. dorei* and *L. symbiosum* co-cultures in inulin and xylan confer various anti-inflammatory and protective effects on model human cell lines.

To evaluate the impact of the supernatants from co-cultures growing on xylan or inulin, anaerobic fermentations were carried out in bioreactors to obtain the former. As expected, inulin cultures showed a higher cell density and faster growth (Fig. S1). Co-cultures in xylan only reached a maximum optical density (OD) of 1.5, and fermentation took longer to reach the stationary phase. In both fermentations, *L. symbiosum* dominated the cultures with up to 90% of total bacteria, as predicted by metabolic modeling (Fig. S1). Acetate and butyrate reached a higher concentration in the inulin co-culture, whereas lactate concentrations were similar in both fermentations (Fig. S2). Propionate and succinate were not detected in the supernatants.

**FIG 8:**
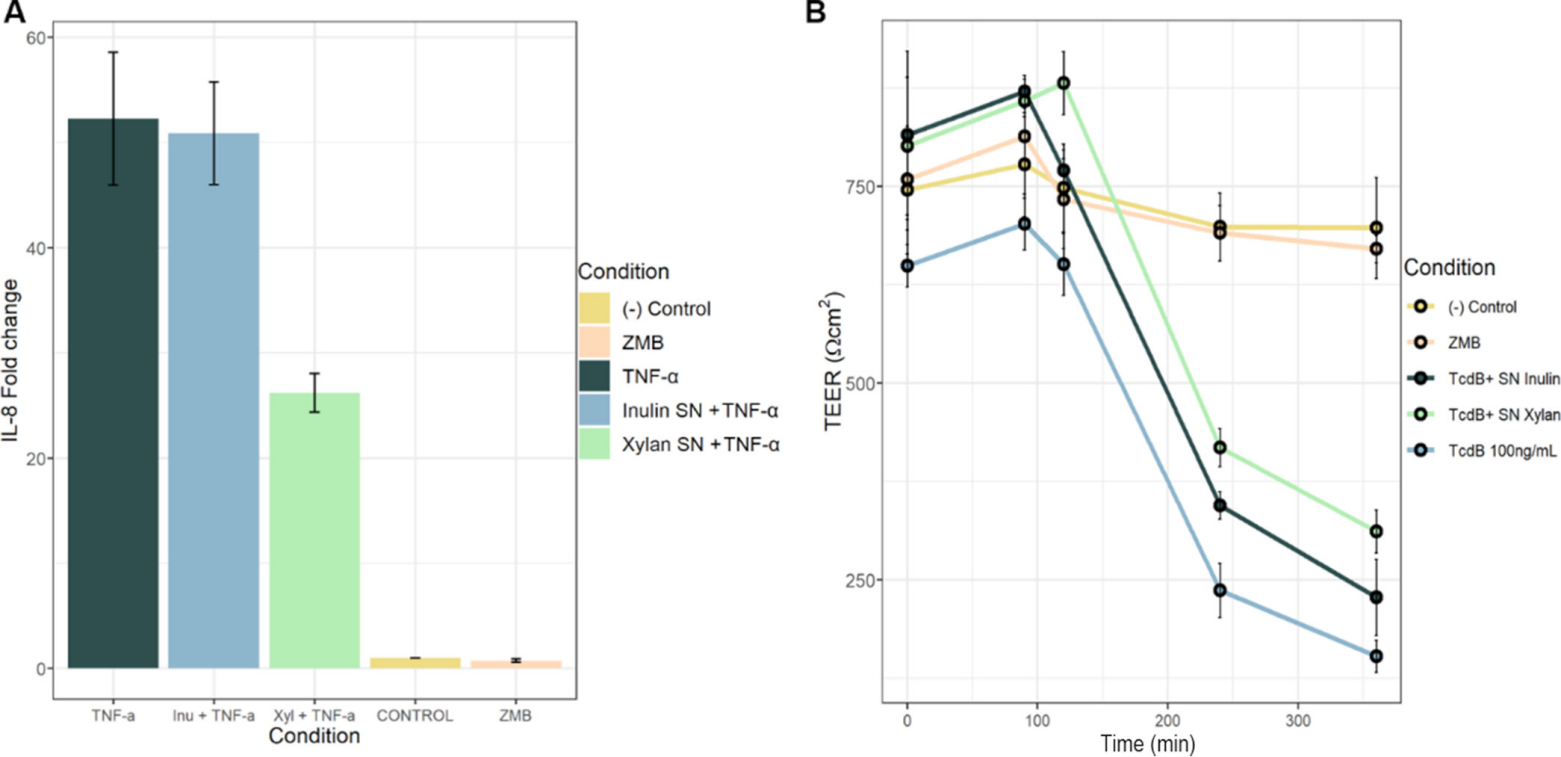
Effect of *L. symbiosum*-*P. dorei* supernatants in cell inflammation and permeability. (A) Inflammatory response of HT-29 Glc^−/+^ cells exposed to TNF-α for 6 h and to *L. symbiosum*-*P. dorei* supernatants. Inflammation was estimated by IL-8 relative expression. Gene expression was normalized to the basal condition (cells with no TNF-α). Bars represent the average ± SD of the fold change in expression of IL-8. (B) Permeability of Caco-2 exposed to TcdB and *L. symbiosum*-*P. dorei* supernatants for 6 h. Dots represent the percentage of the initial TEER value (Ω·cm^2^) in time (average ± SD).

Supernatants were recovered at the end of each fermentation (26 h for inulin and 48 h for xylan), filtered, and used to evaluate their anti-inflammatory and protective potential in human intestinal cell lines. Confluent HT-29 Glc^−/+^ cells were exposed to 25 ng/ml of the pro-inflammatory cytokine tumor necrosis factor-α (TNF-α). This concentration was chosen after an optimization assay (Fig. S3). In a dose-dependent manner, sodium butyrate reduced the interleukin-8 (IL-8) expression induced by TNF-α in HT-29 Glc^−/+^ cells (*P* < 0.05 by *t* test). This effect was abolished at a higher butyrate concentration (30 mM). In this model, adding supernatants from bacteria cultured in xylan exerted an anti-inflammatory effect, as evidenced by a significant 50% reduction in IL-8 expression (Fig. 8A; *P* < 0.05 by *t* test). This effect was not observed with the inulin supernatant (*t* test; *P* > 0.05).

10.1128/msystems.00646-22.3 FIG S3 Evaluation of protective effect of butyrate in model cell lines. (A) Standardization of the intestinal inflammation model in HT-29 Glc^−/+^ cells exposed to increasing concentrations of TNF-α for 6 h. (B) Protective effect of sodium butyrate on HT-29 Glc^−/+^ cells exposed to TNF-α for 6 h. IL-8 expression estimated by q-PCR. (C) Effect of 30 mM butyrate on cells treated or not with TNF-α. The bars represent the average ± SD of the times of change in IL-8 expression relative to baseline (negative control). (D) Effect of TcdB on Caco-2 cell monolayers for 6 h. Time-dependent alteration of transepithelial electrical resistance (TEER) after apical exposure of 100, 250, and 350 ng/ml of TcdB. (E) Protective effect of different concentrations sodium butyrate on Caco-2 cells monolayers exposed to TcdB for 6 h. The points represent the percentage of the initial TEER value over time (average ± SD). A two-way ANOVA test and Bonferroni *post hoc* test were performed to assess statistical significance between the groups (*n* = 3). Download FIG S3, TIF file, 1.4 MB. Copyright © 2022 Hirmas et al. 2022 Hirmas et al. https://creativecommons.org/licenses/by/4.0/ This content is distributed under the terms of the Creative Commons Attribution 4.0 International license.

Finally, confluent Caco-2 cells (transepithelial electrical resistance [TEER] >600 Ω·cm^2^) seeded in a Transwell system were apically exposed to different TcdB concentrations for 6 h (100 to 350 ng/ml; Fig. S3). TcdB is an enterotoxin produced by Clostridioides difficile that disrupts tight junctions in the intestinal epithelium (52). Accordingly, cells exposed to TcdB significantly diminished their TEER values (Fig. 8B; *P* < 0.05 by *t* test). In this model, only a lower butyrate concentration (1.65 mM) showed a significant protective effect, preventing the permeability increase induced by 100 ng/ml of TcdB (Fig. S3; *P* > 0.05 by *t* test). Interestingly, the bacterial supernatants from the xylan fermentation showed a modest but significant protective effect against TcdB action on the epithelial permeability (*P* < 0.05 by *t* test). This effect was not observed for the inulin supernatant with *P. dorei* and *L. symbiosum* (Fig. 8B).

## DISCUSSION

### *In silico* predictions of metabolic interactions using GSMMs.

In this work, we used metabolic models and bidirectional coculturing to analyze the cross-feeding interactions between two gut commensals, *P. dorei* and *L. symbiosum*. The strains used in this study are part of the Human Microbiome Project (53), considered commensals. Still, they have been regarded as markers of type 1 diabetes in children and progression of colorectal cancer, respectively (54, 55).

There was a fairly good agreement between GSMMs predictions and microbial culture assays. GSMMs are extensively curated mathematical models of cellular metabolism (56, 57). Some studies have previously used GSMMs to understand butyrate production in the gut. For example, a mutualistic interaction between Bifidobacterium adolescentis and *F. prausnitzii* was explored *in silico* using GSMMs and validated using *in vitro* models (58). This analysis suggested that acetate was cross-fed between both microorganisms, resulting in butyrate production (58). Similarly, the presented GSMMs provided valuable insights into the mechanisms underpinning the cross-feeding between *P. dorei* and *L. symbiosum*, which could be later verified experimentally. While there are several assumptions made when using these models, some might not be even valid *in vivo* (56), GSMMs are valuable tools for probing the often enormous interaction space and guiding experimentation efforts that enable the formulation and evaluation of more precise hypotheses.

### Mechanism and effects of metabolic cross-feeding in *P. dorei* and *L. symbiosum* cocultures growing on inulin and xylan.

Bidirectional assays provide an experimental platform where both microorganisms grow and interact simultaneously without physical contact. This is key to consider in microbial interactions and different from other setups. Unidirectional assays provide all metabolites left by one microorganism to another, which might contain molecules that would be of use but also several inhibitors. Exchanged metabolites are also produced dynamically, which could explain the different stimulatory effects of *P. dorei* on *L. symbiosum* in uni and birectional assays. The plates used in bidirectional assays provide a separation between bacteria that allows the exchange of multiple metabolites reaching, in theory, chemical equilibrium. We indirectly observed that inulin readily equilibrated on both sides of the membrane, but only certain xylan fractions did.

*L. symbiosum* appeared to increase its fitness by the presence of *P. dorei*, with the latter being unaffected. *P. dorei* acted as a primary degrader of dietary fibers, releasing monosaccharides and organic acids used by *L. symbiosum*. Commensalism interactions between primary fermenters and butyrate producers are common, and several examples have been described previously (27, 28, 30, 59). While *P. dorei* did not see its growth modified by the presence of *L. symbiosum*, it indeed showed an accelerated substrate consumption (Fig. 5) and evident changes in gene expression (Fig. 7), demonstrating it is not neutral to the presence of *L. symbiosum*.

Xylan is a highly complex polysaccharide, with a few members of the microbiota being able to degrade it (60). Moreover, its degradation process is complex and dynamic, as shown in *B. ovatus* (13). Inulin is a more accessible and simple chain of fructose (7). We observed that *P. dorei* grew well in inulin and xylan, supporting the growth of *L. symbiosum* in coculture. As *L. symbiosum* has a limited ability to utilize inulin and rather consumes the smaller chains present in the medium, the results here suggest it benefits from smaller fructans produced by *P. dorei* crossing through the membrane. This result was supported by the overexpression of β-fructofuranosidases in both species (Fig. 7). In contrast, *L. symbiosum* does not have the machinery for xylan or xylose fermentation as opposed to *P. dorei*, which released xylan-degradation products.

Interestingly, cocultures on both substrates resulted in an accelerated substrate consumption (Fig. 5). Competition for resources between both bacteria could help to explain the increased expression of consumption pathways where each bacterium is more efficient. Bacteroides thetaiotaomicron expands the range of carbohydrates it can consume in the presence of Bifidobacterium longum in an animal model (61). Other *Bifidobacterium* species appear to be competitive against B. thetaiotaomicron during inulin consumption, but this effect depends on the length of the inulin chain (62). Interactions shown here are contact independent, indicating they rely exclusively on metabolic exchange.

Xylan TLC plates showed no degradation products in coculture, and xylan utilization genes in *P. dorei* were overexpressed in the xylan coculture. This indicated that xylan was consumed by the enhanced activity of both microorganisms. For instance, Bacteroides ovatus shows a cooperative behavior during xylan utilization, providing xylan degradation products for other gut microbes while benefiting from the microbial community (25). It is likely that *P. dorei* engages in a similar interaction as shown in this study.

Lactate and succinate concentrations in bidirectional culture assays suggested cross-feeding interactions between *P. dorei* and *L. symbiosum* in xylan and inulin. Previous studies have reported that some species from *Lachnospiraceae* can consume lactate and acetate to produce butyrate (30). Another finding of interest is the different SCFA profiles obtained after inulin or xylan fermentation by *P. dorei* cocultures. In accordance with previous studies (26), inulin fermentation resulted in higher concentrations of lactate and fructose, but xylan utilization showed enrichment in acetate and succinate (49). These metabolic differences are probably explained by the chemical nature of the monomers of these fibers (fructose versus xylose), which are metabolized by different microbial pathways.

### Metabolic interactions and cross talk with host cells.

Butyrate has received special attention for being the primary energy source of colonocytes and for maintaining intestinal homeostasis (63). It has a considerable physiological relevance, boosting immune responses and exerting anti-inflammatory effects (64). However, it has been described as exerting paradoxical effects that depend mainly on its concentration (65). Butyrate and sodium butyrate are well recognized for decreasing permeability and enhancing the intestinal barrier (66, 67).

The effect of microbial supernatants of butyrate-producing bacteria on inflammatory parameters and intestinal epithelial permeability has been reviewed and the results strengthen these findings (68, 69). Supernatants of microbiota from Crohn’s disease patients supplemented with Butyricicoccus pullicaecorum 25-3T, or a mix of six butyrate-producing bacteria, improved the integrity of the epithelial barrier evaluated through increased TEER and decreased IL-8 expression in Caco-2 cells (69, 70). Strain 25-3T is safe and a next-generation probiotic (71). Butyrate concentrations in the supernatants evaluated in these studies were not high (0.3 to 7.9 mM), suggesting that butyrate is not the only microbial factor that explains the improvement in barrier function.

Similarly, oral treatment with the butyrate-producer Clostridium butyricum supernatant (19.9 mM butyrate) decreased dextran sulfate sodium-induced damage in mice colonic mucosa (72). It has also been shown to enhance intestinal barrier function in antibiotic-associated diarrhea and protect against C. difficile, in an effect partially mediated by the bacteriocin CBP22 (73–75). Thomson et al. (49) observed that the supernatant of *P. dorei* and *L. symbiosum* cultured in xylan (added to 10% vol/vol) reduced IL-8 expression and NF-κB activation. These results are consistent with our data. Our supernatants were added at 10% vol/vol, so the butyrate concentration administered to the cells ranged between 1 and 3 mM.

In a recent study, the addition of 1.5 mM sodium butyrate significantly increased TEER in Caco-2 cell monolayers (67). This would explain the increase in TEER observed as improvements in the integrity of the epithelial barrier and the reduction in IL-8 expression following treatment with sodium butyrate and microbial supernatants grew in the presence of xylan. However, it is important to note that the presence of the supernatants did not result in a major improvement in the integrity of the barrier function. This suggests that butyrate *per se* may not be as effective as the restoration of butyrate-producing bacteria or the relevance of dietary fibers to produce this or other fermentation products. It is important to highlight that in this study, *in vitro* bioreactor samples were taken at the end of the exponential phase. Besides butyrate, supernatants might contain a variety of molecules such as ammonia that might hinder the positive effect of butyrate on epithelial cells. Future studies are needed to elucidate the mechanism of these bacterial supernatants and identify other metabolites or bioactive compounds that exert a protective role in intestinal cells.

### Conclusions.

The metabolic interactions between two gut commensal microorganisms, *P. dorei* and *L. symbiosum*, were studied by applying a combined approach of metabolic modeling and experimental cell culture assays. Experimental results were consistent with metabolic modeling predictions, pointing to cross-feeding of lactate and succinate in co-cultures growing on inulin or xylan. Both bacteria showed different characteristics in single culture and in the presence of the other partner, both in inulin and xylan. These changes were observed in terms of their growth, substrate consumption, gene expression, and SCFA production. This study provides an example of the capabilities of metabolic models for predicting microbial interactions and how these predictions could be tested in an experimental setup that evaluates bidirectional interactions. Finally, this work suggests that metabolites differentially produced in xylan provide a protective effect against toxin damage *in vitro*.

## MATERIALS AND METHODS

### Metabolic models.

Genome-scale metabolic models (GSMMs) were obtained from AGORA v3.0 (37) for *P. dorei* DSM 17855 and *L. symbiosum* WAL-14673 and cured manually with literature data (9, 25, 48, 76–80). For the curation, an initial analysis of the number of metabolites, number of reactions, and quality of the models was made using MEMOTE v0.9.13 (50, 81). MEMOTE is a tool that evaluates the quality of the reconstructions by checking a set of tests related to stoichiometric consistency, annotation, and biomass reaction. After it was checked that both models had a good score on MEMOTE’s test (>90%), a manual curation step was carried out for each model to verify that the main metabolic pathways were incorporated. We also checked special pathways of interest, such as the incorporation and degradation of inulin and xylan by *P. dorei* and the butyrate production pathways of *L. symbiosum*, in accordance with the literature (13, 25, 32, 76). The energetic requirements and the biomass reaction of the original AGORA reconstructions were kept due to the lack of experimental information. The predictive capabilities of the reconstructions were tested by their capacity to utilize known substrates and produce particular fermentation products (Tables S2 to S6). Precision, accuracy, and *F*-score values for model validation were calculated according to Mendoza et al. (82). Finally, various exchange constraints were introduced to the models to simulate growth in the tested conditions. These and the refined models are available in the Supplemental File S1, which contains the employed MATLAB scripts.

### Computational growth simulations.

Flux simulations were performed in MATLAB 2019b using the COBRA Toolbox v3.0 (81) and GUROBI v9.0.3 optimizer. The media used for the simulations were defined based on a modified version of ZMB (mZMB) (83), the medium used in the experiments. mZMB is a complex media, so the uptake rate bounds of minerals, ions, and vitamin compounds for *in silico* simulations were set to values that ensured they were in excess. The bounds definition for the amino acids uptake rates followed a similar approach. Xylan and inulin are polysaccharides without a fixed chain length; therefore, the formulas described in the Virtual Metabolic Human database were employed for simplicity (84). These formulas enabled definition of appropriate uptake reactions for each fiber. The above methodology ensured that growth simulations used inulin or xylan as main carbon source.

### Community modeling.

The metabolic behavior of cocultures was simulated using the SteadyCom algorithm from COBRA Toolbox, which seeks to determine the maximum community steady-state growth rate assuming balanced growth (44). This approach not only enables the computation of the metabolic flux distribution of the community in a defined environment but also calculates the relative abundance of each species. Importantly, by means of computing the metabolic exchanges for each microorganism (a subset of the metabolic fluxes), we can readily identify cross-feeding metabolites in the community. To determine the cross-feeding direction, Flux Variability Analysis (46) and Monte Carlo random sampling (47) of the steady-state flux space were employed. The first method determines the range of flux variation under a given (sub)optimal state. The second method generates a random sample of the flux solution space of a linear model defined over a convex region, here defined by the mass balances and capacity constraints in the community. In the context of the SteadyCom formulation, once the community growth rate is known, the mathematical representation of the community optimization problem becomes linear and it is suitable for the above method. Monte Carlo sampling was performed generating 200,000 random flux solution points choosing 1 point every 100 steps (thinning). Finally, an exchanged metabolite was deemed to be consumed if the median flux distribution was significantly less than zero. For exchanged metabolites being produced, the opposite was true.

### Strains and culture media.

*Phocaeicola dorei* 5_1_36 and Lachnoclostridium symbiosum WAL-14673 were obtained from BEI Resources (HM-29 and HM-319). For inoculations and initial tests, both microorganisms were cultured in Reinforced Clostridium Media (RCM; Becton, Dickinson, Franklin Lakes, NJ) supplemented with 0.5 g/liter of l-cysteine (RCM-cys; Loba Chemie, India). All incubations were performed at 37°C for 24 to 48 h in an anaerobic jar (Anaerocult; Merck, Darmstadt, Germany) with anaerobic packs (Gaspak EM; Becton, Dickinson, Franklin Lakes, NJ, USA). mZMB medium was prepared as in reference 48.

### Monoculture assays.

Single cultures of both microorganisms were carried out in 96-well plates to evaluate the consumption of different metabolites, such as inulin, xylan, succinate, and lactate. As mentioned before, microorganisms were reactivated for 48 h as mentioned before, in 4 ml of RCM-cys at 37°C under anaerobic conditions. They were centrifuged at 5,000 × *g* and washed with blank prereduced mZMB media. Each bacterium was inoculated in 1 ml mZMB with 2% inulin (Piping Rock, Ronkonkoma, NY) or 2% xylan from birchwood (Sigma-Aldrich, St. Louis, MO, USA). Substrates were previously filtered using 0.22-μm filters (Jet Biofil, China), and microorganisms were inoculated at 5% vol/vol. Samples were transferred to 96-well plates, covered with a mineral oil layer, and cultured in triplicates for 48 h. OD_600_ was measured in a Synergy H1 spectrophotometer (BioTek, Agilent Technologies) at 0, 24 and 48 h. Similarly, each microorganism was cultured in 4 ml of mZMB supplemented with 2% xylan or 2% inulin for 48 h and anaerobic conditions. Tubes were centrifuged at 10.000 × *g* for 5 min, and each supernatant was recovered and filtered as above. 100 μl of each supernatant were added to a well in a 96-well plate that had 100 μl of prereduced mZMB, resulting in a 1:1 mixture. Then, each well with supernatant from one microorganism was inoculated with the other at 5% vol/vol for 24 and 48 h. Absorbance was measured at 600 nm in a Synergy H1 spectrophotometer (BioTek, Agilent Technologies). A paired Student’s *t* test assuming equal variances was performed on the maximum OD value for each condition.

### Bidirectional assays.

Both bacteria were cultured in Tissue Culture Plate Inserts (JetBiofil, China; Fig. 1). *L. symbiosum* was always cultured in the upper insert and *P. dorei* in the lower well. The bacteria were separated by the permeable membrane of the insert (0.1 μm), which allowed the passage of small carbohydrates and metabolites. Cocultures were performed in 250 μl mZMB in the insert and 1 ml in the bottom well. Nine conditions of mono and coculture were tested in duplicate, using 2% of lactose (Sigma-Aldrich), inulin (Piping Rock, Ronkonkoma, NY), or xylan from birchwood (Sigma-Aldrich, St. Louis, MO, USA) as the sole carbon source. The plate was preincubated in an anaerobic jar under anaerobic conditions at room temperature for 48 h to reduce the medium.

Bacteria were reactivated in RCM for 48 h, centrifuged at 10,000 × *g* for 1 min, and washed with blank mZMB. Inoculations were performed at 5% vol/vol. The plates were incubated in an anaerobic jar with anaerobic packs at 37°C for 24 and 48 h. OD_600_ was measured at 0, 24, and 48 h by resuspending the content of each well or insert and transferring 200 μl to a new 96-well plate. Measurements were carried out in a Synergy H1 spectrophotometer (BioTek, Agilent Technologies). Later, samples were transferred to Eppendorf tubes and centrifuged at 10,000 × *g* for 2 min, and the supernatant was separated from pellets. Both were stored at −80°C until use. For the final OD values, the mean of each basal medium without bacteria was subtracted according to the carbon source. A paired Student’s *t* test assuming equal variances was performed on the maximum OD value for each condition.

### Carbohydrate profiling and consumption.

Thin-layer chromatography (TLC) was performed in silica gels F-60 (Merck, Germany), using 1-butanol/ethanol/water 10:8:5 vol/vol as running buffer and 1% orcinol in 10% H_2_SO_4_ in ethanol as the detecting reagent (48, 85). Two microliters of each sample were spotted in the plates, and the chromatogram was developed in one run and left to dry. After the detector was poured and dried, the silica gel was heated at 100°C until the bands were visually detectable. Carbohydrates were also quantified using the phenol-sulfuric acid method (86), quantifying the total amounts of fructose and xylose from inulin and xylan in the supernatants. A paired Student’s *t* test assuming equal variances was performed on the carbohydrate concentrations.

### SCFA quantification.

Acetic, butyric, lactic, propionic, and succinic acids of select supernatants and bioreactor samples from 26 and 48 h were measured in a Lachrom liquid chromatograph (Merck-Hitachi), using an Aminex HPX-87H Ion exclusión column (300 mm × 7.8 mm; Bio-Rad). Thirty microliters of supernatants was injected at a flux of 0.45 ml/min, at 35°C for 35 min. Standard curves were created by measuring nine dilutions of 30 g/liter to 0.155 g/liter of each acid in HPLC grade water. Ten conditions plus a sample of mZMB at hour 48 were tested in biological duplicates. The control of the chromatograph and data analysis was done using Multi-HSM Manager software (Hitachi). A paired Student’s *t* test assuming equal variances was performed on the SCFA data.

### RNA extraction.

RNA was extracted from the pellets of the samples analyzed by HPLC. Total nucleic acids were extracted using a modified version of the phenol-chloroform/isoamyl alcohol method (87). Purity was determined through a 260/280 absorbance ratio in a Tecan Infinite M200 Pro plate reader (Tecan, Austria). After extraction, samples were immediately treated with DNase I (New England BioLabs) using the manufacturer’s protocol for 15 min.

### Reverse transcription.

RNA was converted into cDNA with the AffinityScript QPCR cDNA Synthesis kit (Agilent Technologies, Texas), using the manufacturer’s protocol and random primers and negative control with free nuclease water (Sigma-Aldrich) and an RNase block control for two of each bacterial sample. The final quality of cDNA was assessed by measuring the 260/280 absorbance ratio in a Tecan Infinite M200 Pro plate reader, and absorbance curves from 230 nm to 300 nm were obtained to rule out chemical contamination. All cDNA samples were stored at −20°C until use.

### Quantitative PCR and gene expression.

Primers for calculating changes in gene expression were designed using Primer-BLAST for select genes (see supplementary material). Six genes were chosen for *L. symbiosum*, related to fructan metabolism and butyrate production pathways. Ten genes were selected for *P. dorei* related to short-chain fatty acids (SCFA) production and inulin and xylan consumption. 16S rRNA genes were used for both microorganisms as the reference gene. Quantitative PCRs (qPCRs) were prepared using the SensiFAST SYBR No-ROX kit (Bioline, USA) under the manufacturer instructions and 1 μl of each cDNA. Amplification was performed in an AriaMx real-time PCR system (Agilent Technologies), using 96-well optical plates MicroAmp Fast Optical (ThermoFisher, USA).

Reactions were carried out in a three-step cycling format, with an initial cycle at 95°C for 2 min, and 40 cycles at 95°C for 5 s (denaturation), 60°C for 10 s (annealing), and 72°C for 10 s (extension). All conditions had two biological replicates with three technical replicates. A standard curve was included by loading 10-fold dilutions of genomic DNA, and a negative control was included for each pair of primers. Threshold cycle (*C_T_*) values and efficiency of the reactions were calculated using the Agilent Aria 1.7 software. Changes in gene expression were calculated using the efficiency-corrected method (88). We separated the effect of the carbon source from the culture with the other bacteria, so the monoculture was used as the basal condition for the cocultures in each carbon source, and the monoculture in mZMB was used as the basal condition for the monocultures. The efficiency of each PCR was obtained from the standard curve and considered equal to all samples for that gene. Technical replicates were averaged, but the ratio was calculated independently for biological replicates and then averaged. The ANOVA test was used to evaluate global significance under all conditions and paired *t* test between each pair of conditions when the ANOVA *P* value was smaller than 0.1.

### Experimental design and bioreactor operation.

Both bacteria were cocultured in a 250-ml Minibio bioreactor connected to a MyControl system (Mini-bio Applikon Biotechnology, Netherlands), using xylan or inulin at 20 g/liter as the sole carbon source. Microorganisms were cultured using mZMB with a fixed pH of 5.5, and each fermentation/condition was made in duplicate. Before inoculation, both microbes were grown individually for 48 h at 37°C in an anaerobic jar in mZMB using lactose (20 g/liter) as carbon source. The optical density at 600 nm (OD_600_) was measured, cultures were centrifuged at 3,000 × *g* for 5 min, and the supernatant was discarded. The pellet was resuspended in the fixed groups of mZMB without a carbon source so that the final OD_600_ was equal to 1 for each microorganism before being inoculated to the bioreactor. The bioreactor was autoclaved for 15 min at 121°C, with a 34.2 g/liter of tryptone (Becton, Dickinson, Franklin Lakes, NJ), 1 g/liter of l-cysteine, and 70 ml of distilled water for each experiment. Xylan (20 g/liter) was also autoclaved in the bioreactor, while inulin (20 g/liter) was sterilized with the remaining components of the medium using 0.22-μm filters and inoculated in the bioreactor after being autoclaved (48). Two-hundred microliters of silicone antifoam (polydimethylsiloxane) and nitrogen (99.99% purity grade) were injected at the beginning of the experiment to control foam levels and to generate an anaerobic environment. Fermentation was carried out for 26 to 48 h for inulin or xylan, respectively, at 37°C and stirring at 90 rpm. Automatic injection of 3 M NaOH and 3 M HCl was used to maintain pH at 5.5. Samples were obtained every 4 h, centrifuged at 10,000 × *g* for 2 min, separated into supernatant and pellet, and stored at −20°C until analysis. Pellets were used for DNA extraction and relative abundance determination, while supernatants were used to quantify SCFA production and cellular assays.

### Determination of relative bacterial abundances.

An adapted phenol-chloroform-isoamyl protocol (87) was used to extract total DNA from cell pellets, which was quantified using a NanoQuant Plate in a Tecan Infinite M200 Pro microplate reader. Samples were diluted to 10 ng/μL, and relative abundance was determined by qPCR, with specific primers and protocol previously defined (48). Each sample was quantified in triplicate, and raw data were analyzed using Agilent Aria 1.7 software (Agilent). *C_T_* values were converted into genome copy numbers per ml as in reference 89.

### Cell culture and inflammation assays.

The anti-inflammatory effect of cell supernatants was evaluated in the human colonic adenocarcinoma cell line HT-29 Glc^−/+^ (90). This culture type is created from an HT-29 cell culture that grows in a medium without glucose during 36 passages, and then glucose is added, developing a colonic-type differentiation (91). Cells were grown in 25-cm^2^ flasks using 5 ml of Dulbecco’s modified Eagle’s medium (DMEM), supplemented with 10% vol/vol heat-inactivated fetal bovine serum and 1% penicillin/streptomycin (HyClone, USA). Cells were cultured at 37°C in a humidified incubator with 5% CO_2_ and were subcultured using 0.05% trypsin at 60% to 90% confluence. To standardize the inflammation model, HT-29 Glc^−/+^ cells (passages 47 to 50) were seeded at 4 × 10^5^ cells per well in a 24-well polystyrene plate. When cells reached confluence, TNF-α (R&D Systems, Inc., USA) was added at final concentrations of 0.5, 2.5, 5, 10, 25, and 50 ng/ml. Cultures were incubated for 6 h. Subsequently, cells were washed with phosphate buffer saline (1× PBS, Corning), rapidly detached from the plate with 1× trypsin, and centrifuged at 13,000 × *g* for 2 min at 4°C. Cell pellets were preserved in RNA-Later (Sigma-Aldrich, USA) at 4°C until use.

To measure the protective effect of sodium butyrate, cells in passages 51 to 54 were treated with 25 ng/ml of TNF-α and sodium butyrate (Sigma-Aldrich) at final concentrations of 3.25, 7.5, 15, and 30 mM for 6 h. After incubation, cells were washed with PBS, trypsinized, centrifuged at 13,000 × *g* for 2 min at 4°C, and pellets were preserved in RNA-later until use. As high concentrations of butyrate stimulate IL-8 expression, cells exposed to 30 mM sodium butyrate were used as a positive control, while cells untreated with TNF-α were used as the negative control.

To evaluate the anti-inflammatory effect of the bioreactor supernatants, cells in passages 55 to 59 were seeded at 3 × 10^5^ cells per well in a 48-well plate. Confluent cells were treated with 25 ng/ml of TNF-α and 30 μl of the supernatant of each bioreactor (10%). After 6 h of incubation, cells were separated and preserved as described before.

### Relative gene expression in HT-29 cells.

Total RNA from HT-29 Glc^−/+^ cells was extracted using E.Z.N.A. total RNA kit (Omega Bio-Tek, USA), according to the manufacturer’s protocol and reference 49. Each sample was treated with DNA-Free DNase Treatment and Removal Reagents kit (Ambion, Life Technologies) and transcribed to cDNA using the High-Capacity cDNA Reverse Transcription kit (Applied Biosystems). Real-time PCR (qPCR) was used to quantify the expression of the IL-8 gene as a marker of inflammation. Brilliant II SYBR Green QPCR Master Mix (Agilent Technologies) was used with a total reaction volume of 20 μl, using an AriaMx Realtime PCR System (Agilent Technologies). Samples were initially incubated for 2 min at 50°C and later 2 min at 95°C. Amplification consisted of 40 cycles of 95°C for 3 s, 63°C for 30 s, and 20 s at 72°C. A final extension cycle was included at 95°C 3 s, 63°C 30 s, and 95°C 30 s. Similar to as described above, *C_T_* values were automatically determined, and changes in gene expression were determined by the Relative Standard Curve method (88). Serial dilutions (1:10) of 10 ng/ml of cDNA of cells unexposed to TNF-α (negative control) were used to create relative standard curves for IL-8 (target gene) and β-actin (ACTB) (endogenous control, housekeeping gene). Primer sequences for IL-8 and β-actin were obtained from references 92, 93. All experiments were performed in triplicate, and the final results were expressed as IL-8/ACTB and normalized by the basal condition.

### Permeability assays.

Permeability assays were carried out in Caco-2 cells, considering its similitude to the small intestine epithelium and its high transepithelial electrical resistance (TEER) (>500 to 600 Ohm·cm^2^) (94). Cells were grown in 25-cm^2^ flasks using 5 ml of DMEM/F12 (Gibco, USA) supplemented with 10% vol/vol heat-inactivated fetal bovine serum (Gibco, USA), 1% penicillin/streptomycin (HyClone, USA) and 1% (vol/vol) of nonessential amino acids (Corning). Cells were cultured at 37°C in a humidified incubator with 5% CO_2_ and then subcultured using 0.05% trypsin at 60% to 90% confluence. For standardization, Caco-2 cells (passages 16 to 19) were cultured at a density of 1 × 10^5^ cells per well in polycarbonate Transwell filters of 12-mm diameter and 0.4-μm pore size (Costar 3460, Corning), previously treated with rat tail collagen (Gibco). Apical and basolateral compartments were filled with 1.5 and 0.5 ml of medium, respectively. The medium was changed three times per week until cells were differentiated (15 to 21 days). When basal TEER reached values higher than 600 Ω·cm^2^, the apical medium was removed, and TcdB toxin was added to the medium without inactivated fetal bovine serum, at final concentrations of 100, 250, and 350 ng/ml. Monolayers were incubated for 6 h with regular measurement of the TEER.

To evaluate the protective effect of sodium butyrate, monolayers of Caco-2 were exposed for 6 h to 100 ng/ml of TcdB and sodium butyrate at final concentrations of 1.65, 3.25, 7.5, 15, and 30 mM. Cells unexposed to TcdB and cells exposed to 30 mM sodium butyrate were used as the negative and positive control, respectively. The TEER was regularly measured at 0, 90, 120, 240, and 360 min during the experiment. The protective effect of the supernatants from inulin and xylan bioreactors was evaluated using monolayers of Caco-2 cells (passages 24 to 28) treated apically with 100 ng/ml of TcdB (10%) of the supernatant of the reactors that showed a good anti-inflammatory response. Cells unexposed to TcdB and cells exposed to 10% of mZMB were used as controls. The TEER was regularly measured, as mentioned below.

### TEER measurements.

Transepithelial electrical resistance was measured according to reference 95, using an ohm/voltmeter (EVOM2, WPI) connected to Ag-AgCl electrodes. Briefly, a monolayer of Caco-2 cells was grown in Transwell plates, and two electrodes were located on each side of the monolayer. A current was transmitted through the monolayer, and the voltage difference and resistance were measured. The voltage difference is a measurement of the effectiveness of the monolayer as a protective barrier, reflecting its integrity. According to preliminary tests, cells were considered confluent when the baseline TEER was higher than 600 Ω·cm^2^. The results of TEER (Ω·cm^2^) are expressed as percentages of the initial value: TEER = (TEERexp × 100)/TEERbasal.

### Data availability.

All raw data, code, tables, calculations, and primer sequences are included in the supplemental material.
