# Supplementary material for: Metabolic Modeling and Bidirectional Culturing of Two Gut Microbes Reveal Cross-Feeding Interactions and Protective Effects on Intestinal Cells
Source: mSystems. 2022 Aug 25;7(5):e00646-22. doi: 10.1128/msystems.00646-22 (PMC9600892; doi:10.1128/msystems.00646-22)
Supplement: TABLE S1 [file msystems.00646-22-s0004.pdf]

Supplementary Tables

Table S1. Modifications introduced to the AGORA reconstructions of P. *dorei* and *L. symbiosum*

Model[1]	VMH ID	Reaction name	Reaction formula	VMH Subsystem	Associated gene ID	Comments	References
Pd	ALAt2r	L-Alanine Reversible Transport via Proton Symport	ala_L[e] + h[e] -> ala_L[c] + h[c]		556260.3.peg.4078	Based on Transport DB information and genome annotation.	
Pd	EX_ala_L(e)	Exchange of L-Alanine	ala_L[e] <=>	Exchange/demand reaction		Needed to keep the balance in the metabolic network reconstruction.	
Pd	AMAA	N-acetylmuramoyl-L-alanine amidase	acmama[c] + h2o[c] -> acmam[c] + ala_L[c]	Cell wall biosynthesis	556260.3.peg.1434	Based on comparative genomics with Bacteroides strains modeled in AGORA.	
Pd	ARGSS	Argininosuccinate Synthase	asp_L[c] + atp[c] + citr_L[c] -> amp[c] + argsuc[c] + h[c] + ppi[c]	Alanine and aspartate metabolism	556260.3.peg.1880	Based on comparative genomics with Bacteroides strains modeled in AGORA.	
Pd	DHFOR2	Dihydrofolate reductase	dhf[c] + nadp[c] <=> fol[c] + nadph[c]	Folate metabolism	556260.3.peg.3768	Based on comparative genomics with Bacteroides strains modeled in AGORA.	
Pd	DHFR	Dihydrofolate Reductase	dhf[c] + h[c] + nadph[c] <=> nadp[c] + thf[c]	Folate metabolism	556260.3.peg.3768	Based on comparative genomics with Bacteroides strains modeled in AGORA.	
Pd	CYSTL	Cystathionine b-lyase	cyst_L[c] + h2o[c] -> hcys_L[c] + nh4[c] + pyr[c]	Methionine and cysteine metabolism		Based on comparative genomics with Bacteroides strains modeled in AGORA.	
Pd	ACNAMS	N-acetylneuraminate synthase	acmana[c] + h2o[c] + pep[c] -> acnam[c] + pi[c]	Aminosugar metabolism	556260.3.peg.1419	Based on comparative genomics with Bacteroides strains modeled in AGORA.	
Pd	EX_xylan(e)	Xylan exchange	xylan[e] <=>			Needed to keep the balance in the metabolic network reconstruction.	
Pd	XYLAN_DEGe	Xylan degradation, assumed extracellular	527.0 h2o[e] + xylan[e] -> 528.0 xyl_D[e]			A simplified version of the reaction catalyze by the endo -1,4- beta xylanase (EC 3.2.1.8) . The Pd genome assembly (Genbank: GCA_000158335.2) contents the gene BSEG_01391 (Uniprot KB C3R891) identified as beta-xylanase and previous experiments in our group suggests Pd growth in this carbon source (Thomson et al, 2018). The endo -1,4- beta xylanase is an extracellular enzyme.	Thomson, P., Medina, D. A., Ortúzar, V., Gotteland, M., & Garrido, D. (2018). Anti-inflammatory effect of microbial consortia during the utilization of dietary polysaccharides. Food Research International, 109, 14-23.
Ls	XYLI1	Xylose isomerase	xyl_D[c] <=> xylu_D[c]			Xylose isomerase is an important enzyme for the pentose sugar utilization. The gene HMPREF9475_02901 from the Ls assembly (Genome ID: 742741.3, PATRIC database) encodes a predicted xylose isomerase inferred by homology. Several species of the Clostridium genus posses this enzyme (Moes et al 1996, Glimmer et al 2010), including the Clostridium symbiosum ATCC 14940 strain (Genome ID: 411472.5, PATRIC).	Moes, C. J., Pretorius, I. S., & Van Zyl, W. H. (1996). Cloning and expression of the Clostridium thermosulfurogenes D-xylose isomerase gene (xylA) in Saccharomyces cerevisiae. Biotechnology letters, 18(3), 269-27<br><br>Grimmler, C., Held, C., Liebl, W., & Ehrenreich, A. (2010). Transcriptional analysis of catabolite repression in Clostridium acetobutylicum growing on mixtures of D-glucose and D-xylose. Journal of biotechnology, 150(3), 315-323
Pd	INULINASE	Inulin degradation by beta-2->1-fructanase	29.0 h2o[c] + inulin[c] -> 29.0 fru[c] + glc_D[c]			Previous experiments in our group suggests Pd growth in this carbon source (Thomson et al, 2018). Genomic analysis indicates the presence of multiple GH32 enzymes associated with the fructans consumption, and in particular, the EC:3.2.1.80 enzyme. For simplicity, we modelled the inulin comsuption with an intracellular reaction from VMH.	Thomson, P., Medina, D. A., Ortúzar, V., Gotteland, M., & Garrido, D. (2018). Anti-inflammatory effect of microbial consortia during the utilization of dietary

							polysaccharides. Food Research International, 109, 14-23.
Pd	INULINabc	Inulin import through ABC transport system	atp[c] + h2o[c] + inulin[e] -> adp[c] + h[c] + inulin[c] + pi[c]			Previous experiments in our group suggests Pd growth in this carbon source (Thomson et al, 2018). Genomic analysis indicates the presence of multiple GH32 enzymes associated with the fructans consumption, and in particular, the EC:3.2.1.80 enzyme. For simplicity, we modelled the inulin comsuption with an ABC transporter.	Thomson, P., Medina, D. A., Ortúzar, V., Gotteland, M., & Garrido, D. (2018). Anti-inflammatory effect of microbial consortia during the utilization of dietary polysaccharides. Food Research International, 109, 14-23.
Pd	EX_inulin(e)	Inulin exchange	inulin[e] <=>			Needed to keep the balance in the metabolic network reconstruction.	
Ls	INULINASE	Inulin degradation by beta-2->1-fructanase	29.0 h2o[c] + inulin[c] -> 29.0 fru[c] + glc_D[c]			The only report of Ls culture experiments indicates that this specie don't grow in inulin (Kaneuchi et al, 1976), however, previous experiments in our group suggests Ls growth in this carbon source (Thomson et al, 2018). Ls is poorly described in literature, so we choose to replicate the set of reactions for inulin consumption to qualitatively model the effect of this carbon source in the co-growth simulations.	KANEUCHI, C., WATANABE, K., TERADA, A., BENNO, Y., & MITSUOKA, T. (1976). Taxonomic Study of Bacteroides clostridiiformis subsp. clostridiiformis (Burri and Ankersmit) Holdeman and Moore and of Related Organisms: Proposal of Clostridium clostridiiformis (Burri and Ankersmit) comb. nov. and Clostridium symbiosum (Stevens) comb. nov. International Journal of Systematic and Evolutionary Microbiology, 26(2), 195-204.<br><br>Thomson, P., Medina, D. A., Ortúzar, V., Gotteland, M., & Garrido, D. (2018). Anti-inflammatory effect of microbial consortia during the utilization of dietary polysaccharides. Food Research International, 109, 14-23.
Ls	INULINabc	Inulin import through ABC transport system	atp[c] + h2o[c] + inulin[e] -> adp[c] + h[c] + inulin[c] + pi[c]			idem as INULINASE reaction.	
Ls	EX_inulin(e)	Inulin exchange	inulin[e] <=>			Needed to keep the balance in the metabolic network reconstruction.	

(1) Abbreviations: Ls: *Lachnoclostridium symbiosum* WAL 14673, Pd: *Phocaeicola dorei* 5_1_36/D4
