# Supplementary material for: Metabolic Modeling and Bidirectional Culturing of Two Gut Microbes Reveal Cross-Feeding Interactions and Protective Effects on Intestinal Cells
Source: mSystems. 2022 Aug 25;7(5):e00646-22. doi: 10.1128/msystems.00646-22 (PMC9600892; doi:10.1128/msystems.00646-22)
Supplement: TABLE S2 [file msystems.00646-22-s0005.pdf]

**Table S2. Experimental evidence and model prediction of substrate consumption for *Lachnoclostridium symbiosum***

| VMH ID | Carbon source | Does it growth *in vivo*? | Reference | Prediction |
|---|---|---|---|---|
| EX_fru(e) | D-fructose | Yes | This study | Yes |
| EX_lcts(e) | Lactose | Yes | This study | No |
| EX_glc_D(e) | D-Glucose | Yes | Kaneuchi et al. 1976[1] | Yes |
| EX_xyl_D(e) | D-Xylose | No | This study | No |
| EX_arab_L(e) | L-arabinose | Yes | Kaneuchi et al. 1976 | Yes |
| EX_rib_D(e) | D-ribose | No | Kaneuchi et al. 1976 | Yes |
| EX_man(e) | D-mannose | Yes | Kaneuchi et al. 1976 | No |
| EX_gal(e) | D-galactose | Yes | Kaneuchi et al. 1976 | No |
| EX_rmn(e) | L-rhamnose | No | Kaneuchi et al. 1976 | No |
| EX_sucr(e) | Sucrose | No | Kaneuchi et al. 1976 | No |
| EX_malt(e) | D-maltose | No | Kaneuchi et al. 1976 | No |
| EX_cellb(e) | Cellobiose | No | Kaneuchi et al. 1976 | No |
| EX_melib(e) | Melibiose | No | Kaneuchi et al. 1976 | No |
| EX_raffin(e) | Raffinose | No | Kaneuchi et al. 1976 | No |
| EX_dextrin(e) | Dextrin | No | Kaneuchi et al. 1976 | No |
| EX_glyc(e) | Glycerol | No | Kaneuchi et al. 1976 | No |
| EX_sbt_D(e) | Sorbitol | No | Kaneuchi et al. 1976 | No |
| EX_salcn(e) | Salicin | No | Kaneuchi et al. 1976 | No |

(1) Kaneuchi, C., Watanabe, K., Terada, A., Benno, Y., & Mitsuoka, T. (1976) Taxonomic Study of Bacteroides clostridiiformis subsp. clostridiiformis (Burri and Ankersmit) Holdeman and Moore and of Related Organisms: Proposal of Clostridium clostridiiformis (Burri and Ankersmit) comb. nov. and Clostridium symbiosum (Stevens) comb. nov. International Journal of Systematic and Evolutionary Microbiology, 26(2), 195-204. https://doi.org/10.1099/00207713-26-2-195
