# Supplementary material for: Metabolic Modeling and Bidirectional Culturing of Two Gut Microbes Reveal Cross-Feeding Interactions and Protective Effects on Intestinal Cells
Source: mSystems. 2022 Aug 25;7(5):e00646-22. doi: 10.1128/msystems.00646-22 (PMC9600892; doi:10.1128/msystems.00646-22)
Supplement: TABLE S3 [file msystems.00646-22-s0006.pdf]

Table S3. Experimental evidence and model prediction of fermentation products in *Lachnoclostridium symbiosum*

VMH ID	Product	Does produce it *in vivo*?	References	Prediction
EX_ac(e)	Acetate	Yes	This study, Thomson et al. 2018[1]	Yes
EX_but(e)	Butyrate	Yes	This study, Thomson et al. 2018, Vital et al. 2014[2]	Yes
EX_for(e)	Formate	Yes	Virtual Metabolic Human	Yes
EX_h2(e)	Hydrogen	Yes	Virtual Metabolic Human	Yes
EX_lac_L(e)	L-Lactate	No	This study, Thomson et al. 2018	Yes
EX_ppa(e)	Propionate	No	This study	No
EX_succ(e)	Succinate	No	This study	Yes

(1) Thomson, P., Medina, D. A., Ortúzar, V., Gotteland, M., & Garrido, D. (2018) Anti-inflammatory effect of microbial consortia during the utilization of dietary polysaccharides. Food Research International, 109, 14-23. https://doi.org/10.1016/j.foodres.2018.04.008
(2) Vital, M., Howe, A., Tiedje, J. (2014) Revealing the Bacterial Butyrate Synthesis Pathways by Analyzing (Meta)genomic Data. mBio, 5(2), e00889-14. https://doi.org/10.1128/mBio.00889-14
