# Supplementary material for: Metabolic Modeling and Bidirectional Culturing of Two Gut Microbes Reveal Cross-Feeding Interactions and Protective Effects on Intestinal Cells
Source: mSystems. 2022 Aug 25;7(5):e00646-22. doi: 10.1128/msystems.00646-22 (PMC9600892; doi:10.1128/msystems.00646-22)
Supplement: TABLE S4 [file msystems.00646-22-s0007.pdf]

Table S4. ***L. symbiosum*** **and** ***P. dorei*** **model evaluation results**

Metric	Ls Value	Pd Value
TP	7	17
TN	12	10
FP	3	1
FN	3	6
Total	25	34.0
Sensitivity	0.70	0.74
Specificity	0.80	0.91
Precision	0.70	0.94
N.P.V	0.80	0.62
Accuracy	0.76	0.79
F-Score	0.70	0.83
