# Supplementary material for: Metabolic Modeling and Bidirectional Culturing of Two Gut Microbes Reveal Cross-Feeding Interactions and Protective Effects on Intestinal Cells
Source: mSystems. 2022 Aug 25;7(5):e00646-22. doi: 10.1128/msystems.00646-22 (PMC9600892; doi:10.1128/msystems.00646-22)
Supplement: TABLE S5 [file msystems.00646-22-s0008.pdf]

Table S5. Experimental evidence and model prediction of substrate consumption of *Phocaeicola dorei*

VMH ID	Carbon source	Does it growth *in vivo*?	Reference	Prediction
EX_inulin(e)	Inulin	Yes	This study	Yes
EX_arab_L(e)	L-Arabinose	Yes	Bakir et al. 2006[1]	Yes
EX_glc_D(e)	D-Glucose	Yes	Bakir et al. 2006	Yes
EX_lcts(e)	Lactose	Yes	Bakir et al. 2006	Yes
EX_malt(e)	D-Maltose	Yes	Bakir et al. 2006	Yes
EX_man(e)	D-Mannose	Yes	Bakir et al. 2006	Yes
EX_raffin(e)	Raffinose	Yes	Bakir et al. 2006	Yes
EX_rmn(e)	L-Rhamnose	Yes	Bakir et al. 2006, Gao et al. 2021[2]	Yes
EX_sucr(e)	Sucrose	Yes	Bakir et al. 2006	Yes
EX_xyl_D(e)	D-Xylose	Yes	Bakir et al. 2006, This study	Yes
EX_xylan(e)	Xylan	Yes	This study	Yes
EX_cellb(e)	Cellobiose	No	Bakir et al. 2006	No
EX_salcn(e)	Salicin	No	Bakir et al. 2006	No
EX_tre(e)	Trehalose	No	Bakir et al. 2006	No
EX_fru(e)	D-Fructose	Yes	Gao et al. 2021	No
EX_acgal(e)	N-Acetylgalactosamine	Yes	Gao et al. 2021	No
EX_acgam(e)	N-Acetyl-D-glucosamine	Yes	Gao et al. 2021	Yes
EX_acnam(e)	N-Acetylneuraminic acid	Yes	Gao et al. 2021	No
EX_arabinogal(e)	Arabinogalactan	Yes	Gao et al. 2021	No
EX_arabinoxyl(e)	Arabinoxylan	Yes	Gao et al. 2021	No
EX_amylopect900(e)	Amylopectin	No	Gao et al. 2021	No
EX_lmn30(e)	Laminarin	No	Gao et al. 2021	No
EX_levan1000(e)	Levan	No	Gao et al. 2021	No
EX_galur(e)	D-Galacturonic acid	No	Gao et al. 2021	Yes
EX_pullulan1200(e)	Pullulan	Yes	Gao et al. 2021	No
EX_mnl(e)	D-Mannitol	No	Bakir et al. 2006	No
EX_glyc(e)	Glycerol	No	Bakir et al. 2006	No
EX_sbt_D(e)	D-Sorbitol	No	Bakir et al. 2006	No

(1) Bakir, M. A., Sakamoto, M., Kitahara, M., Matsumoto, M., & Benno, Y. 2006. (s. f.). Bacteroides dorei sp. Nov., isolated from human faeces. International Journal of Systematic and Evolutionary Microbiology, 56(7), 1639-1643. https://doi.org/10.1099/ijs.0.64257-0
(2) Gao, G., Cao, J., Mi, L., Feng, D., Deng, Q., Sun, X., Zhang, H., Wang, Q., & Wang, J. (2021). BdPUL12 depolymerizes β-mannan-like glycans into mannooligosaccharides and mannose, which serve as carbon sources for Bacteroides dorei and gut probiotics. International Journal of Biological Macromolecules, 187, 664-674. https://doi.org/10.1016/j.ijbiomac.2021.07.172
