# Supplementary material for: Metabolic Modeling and Bidirectional Culturing of Two Gut Microbes Reveal Cross-Feeding Interactions and Protective Effects on Intestinal Cells
Source: mSystems. 2022 Aug 25;7(5):e00646-22. doi: 10.1128/msystems.00646-22 (PMC9600892; doi:10.1128/msystems.00646-22)
Supplement: TABLE S6 [file msystems.00646-22-s0009.pdf]

Table S6. Experimental evidence and model prediction of fermentation products in *Phocaeicola dorei*

VMH ID	Product	Does produce it *in vivo*?	References	Prediction
EX_ac(e)	Acetate	Yes	This study, Thomson et al. 2018[1]	Yes
EX_h2(e)	Hydrogen	Yes	Chassard et al. 2007[2]	Yes
EX_lac_D(e)	D-Lactate	Yes	Thomson et al. 2018, This study	Yes
EX_ppa(e)	Propionate	Yes	This study	Yes
EX_succ(e)	Succinate	Yes	This study	Yes
EX_but(e)	Butyrate	No	This study, Thomson et al. 2018	No

(1) Thomson, P., Medina, D. A., Ortúzar, V., Gotteland, M., & Garrido, D. (2018) Anti-inflammatory effect of microbial consortia during the utilization of dietary polysaccharides. Food Research International, 109, 14-23. https://doi.org/10.1016/j.foodres.2018.04.008
(2) Chassard, C., Goumy, V., Leclerc, M., Del'homme, C., & Bernalier-Donadille, A. (2007). Characterization of the xylan-degrading microbial community from human faeces: Xylanolytic microbiota from human faeces. FEMS Microbiology Ecology, 61(1), 121-131. https://doi.org/10.1111/j.1574-6941.2007.00314.x
