# Supplementary material for: Metabolic Modeling and Bidirectional Culturing of Two Gut Microbes Reveal Cross-Feeding Interactions and Protective Effects on Intestinal Cells
Source: mSystems. 2022 Aug 25;7(5):e00646-22. doi: 10.1128/msystems.00646-22 (PMC9600892; doi:10.1128/msystems.00646-22)
Supplement: TABLE S7 [file msystems.00646-22-s0010.pdf]

Table S7. Memote model evaluation results

Criteria	Pd[1] Achievement percentage (%)	Ls[1] Achievement percentage (%)
Consistency	95.53	92.44
Annotation of metabolites	24.39	24.92
Annotation of reactions	49.36	48.4
Annotation of genes	0	0
Annotation of SBO terms	0	0

(1) Abbreviations: Ls: *Lachnoclostridium symbiosum* WAL 14673, Pd: *Phocaeicola dorei* 5_1_36/D4
