# Supplementary figures and images for: Metabolic Modeling and Bidirectional Culturing of Two Gut Microbes Reveal Cross-Feeding Interactions and Protective Effects on Intestinal Cells
Source: mSystems. 2022 Aug 25;7(5):e00646-22. doi: 10.1128/msystems.00646-22 (PMC9600892; doi:10.1128/msystems.00646-22)

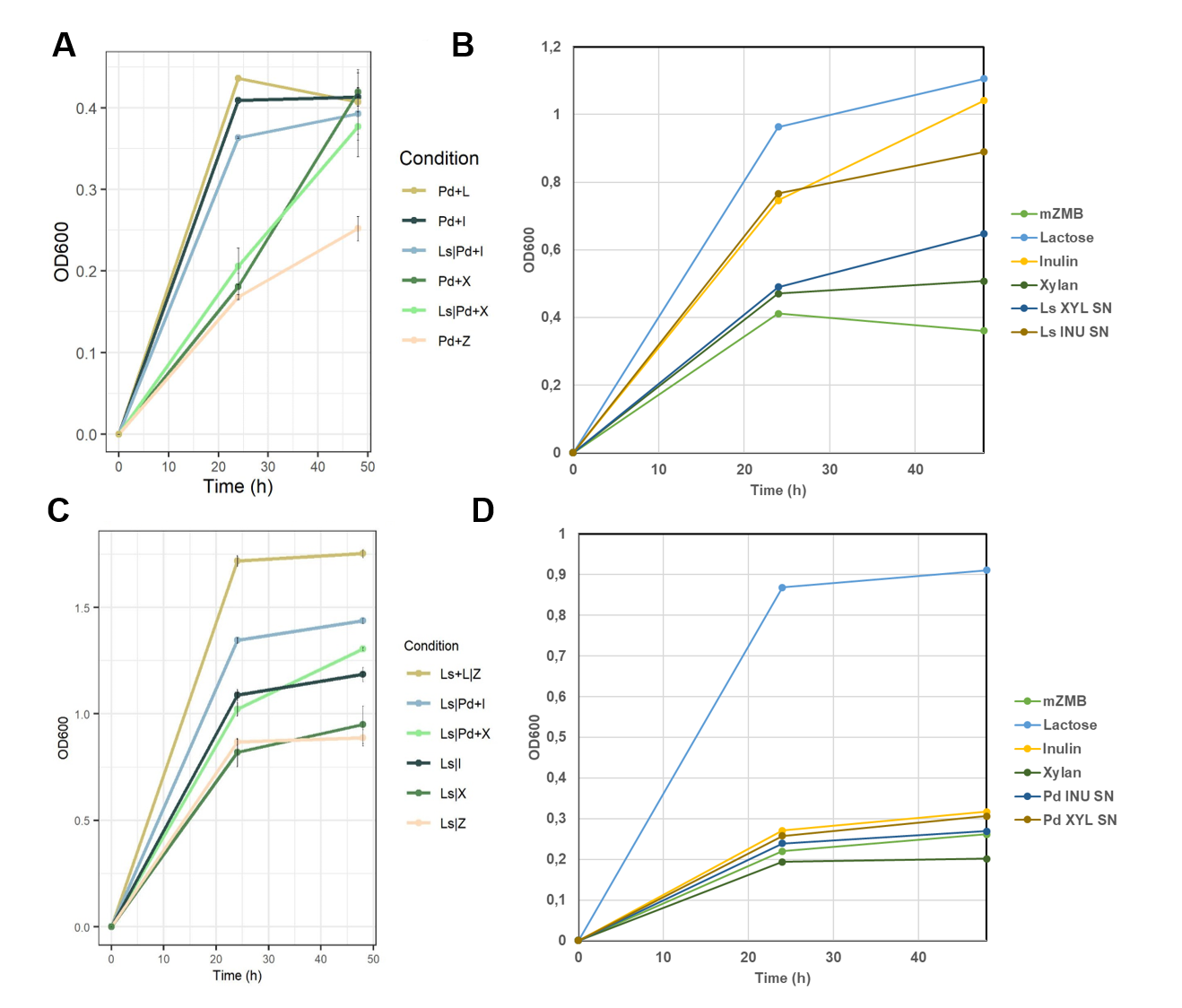

Supplement: FIG S2 [file msystems.00646-22-s0001.tif]

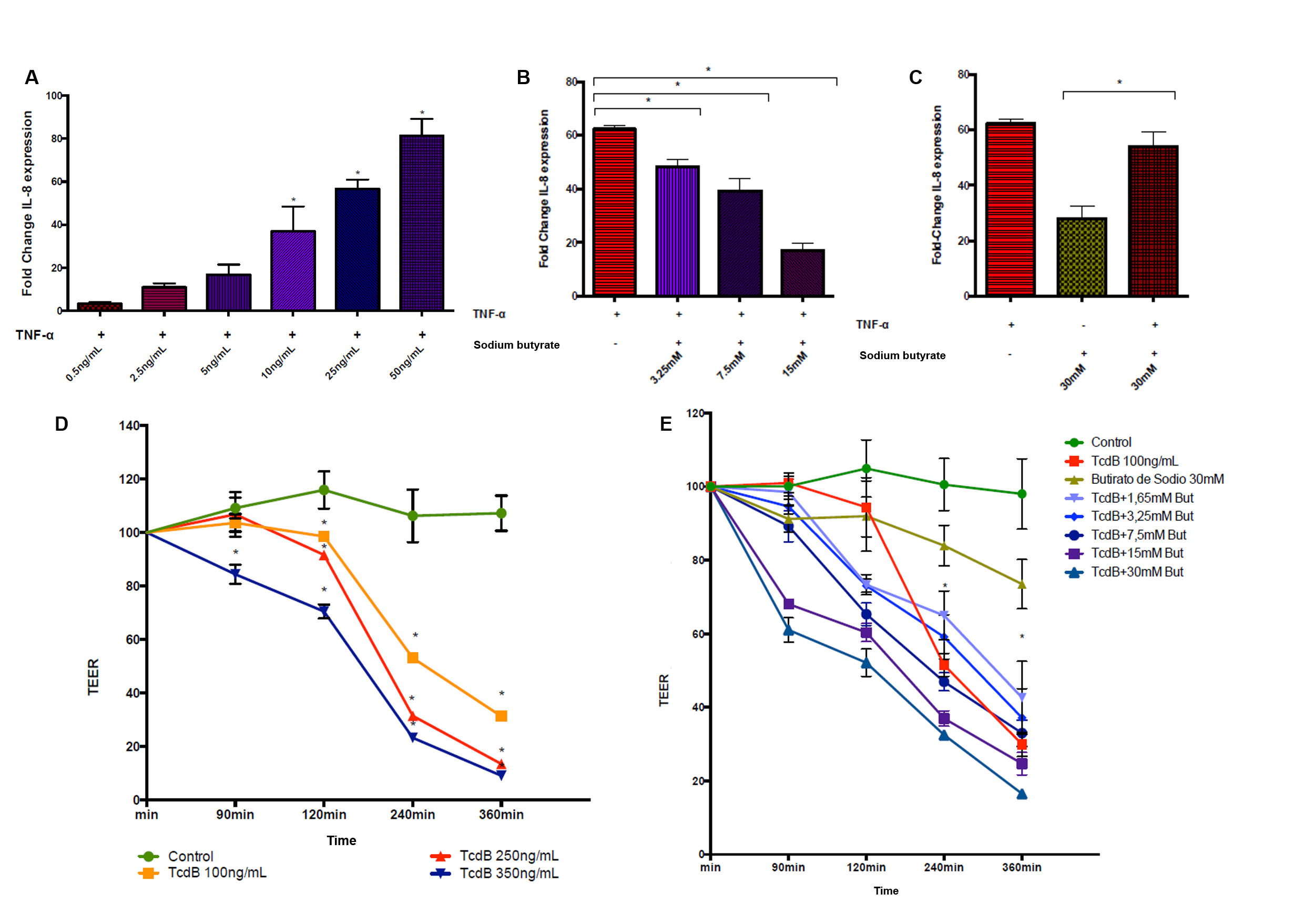

Supplement: FIG S3 [file msystems.00646-22-s0003.tif]
